# Advances in Development of Antimicrobial Peptidomimetics as Potential Drugs

**DOI:** 10.3390/molecules22091430

**Published:** 2017-08-29

**Authors:** Natalia Molchanova, Paul R. Hansen, Henrik Franzyk

**Affiliations:** Department of Drug Design and Pharmacology, Faculty of Health and Medical Sciences, University of Copenhagen, Jagtvej 162, DK-2100 Copenhagen, Denmark; natalia.molchanova@sund.ku.dk

**Keywords:** antimicrobial peptides, peptidomimetics, peptoids, antibiotics

## Abstract

The rapid emergence of multidrug-resistant pathogens has evolved into a global health problem as current treatment options are failing for infections caused by pan-resistant bacteria. Hence, novel antibiotics are in high demand, and for this reason antimicrobial peptides (AMPs) have attracted considerable interest, since they often show broad-spectrum activity, fast killing and high cell selectivity. However, the therapeutic potential of natural AMPs is limited by their short plasma half-life. Antimicrobial peptidomimetics mimic the structure and biological activity of AMPs, but display extended stability in the presence of biological matrices. In the present review, focus is on the developments reported in the last decade with respect to their design, synthesis, antimicrobial activity, cytotoxic side effects as well as their potential applications as anti-infective agents. Specifically, only peptidomimetics with a modular structure of residues connected via amide linkages will be discussed. These comprise the classes of α-peptoids (*N*-alkylated glycine oligomers), β-peptoids (*N*-alkylated β-alanine oligomers), β^3^-peptides, α/β^3^-peptides, α-peptide/β-peptoid hybrids, α/γ *N*-acylated *N*-aminoethylpeptides (AApeptides), and oligoacyllysines (OAKs). Such peptidomimetics are of particular interest due to their potent antimicrobial activity, versatile design, and convenient optimization via assembly by standard solid-phase procedures.

## 1. Introduction

Antimicrobial resistance (AMR) is a global health-care problem causing the death of nearly 700,000 people each year [1], and predictions indicate that this number may reach up to 10 million deaths annually by 2050. The annual cost of AMR is estimated at €1.5 billion in the EU and US$5 billion in the USA [2]. According to the Infectious Diseases Society of America (IDSA), the most worrying bacteria comprise the multidrug-resistant (MDR) ESKAPE pathogens *Enterococcus faecium*, *Staphylococcus aureus*, *Klebsiella pneumoniae*, *Acinetobacter baumannii*, and *Enterobacter* species, which together cause the majority of US hospital infections. Furthermore, resistance to the last-resort antibiotics colistin and carbapenems for treatment of difficult Gram-negative infections is spreading rapidly. For example, shortly after the emergence of a mobilized colistin resistance gene (mcr-1) in bacteria from pigs in China in 2015, it was rapidly distributed across Europe and to Canada and USA [3]. The mcr-1 gene is the first polymyxin resistance-inducing gene capable of horizontal transfer, implying that it may spread among different bacteria, such as *Escherichia coli*, *P. aeruginosa* and *K. pneumoniae*. In 2016, after the initiation of screening for bovine and porcine colistin-resistant *E. coli*, a new resistance gene termed mcr-2 was identified in Belgium mcr-2 [4]. Carbapenem antimicrobial resistance, mediated by transferable carbapenemase-encoding genes and efflux pumps, has been found on all continents [5]. Also, a recent review of currently available data reports on alarming rates of carbapenem resistance in *Pseudomonas* spp. and *Acinetobacter* spp. [6].

Multidrug resistance in Gram-positive bacteria such as methicillin-resistant *Staphylococcus aureus* (MRSA), vancomycin-resistant enterococci (VRE), and *Clostridium difficile* remains a challenge in infectious diseases. Community-associated MRSA strains, which cause serious skin and soft tissue infections in otherwise healthy individuals, have emerged [7]. Infectious diarrhea in hospitalized patients caused by *Clostridium difficile* [8], and a high incidence of VRE colonisation among children with hematological/oncological diseases [9], both constitute severe health issues.

Human medicine is mainly concerned with AMR emerging in human pathogens, and thus it is often overlooked that companion animals (dogs, cats, horses etc.) and food-producing animals (cattle, pigs, sheep, chickens etc.) are also treated with related antibiotics. Therefore, the veterinary area may also contribute to development of resistant bacterial pathogens. In fact, two thirds of the consumption of antibiotics in the EU and 70% of their usage in the US occurs in the animal farming industry [10]. In many countries, even colistin is administered to pigs, veal calves, and poultry to treat and prevent diarrhea caused by *E. coli* and *Salmonella* spp. [11]. Administration of tetracyclines to food animals have resulted in the spreading of the livestock-associated methicillin-resistant *S. aureus* CC398 (LA-MRSA CC398) [12].

Furthermore, pet animals are often treated with the same types of antibiotics as humans, thereby enhancing the risk of AMR development in humans [13]. The importance of proper antibiotic use in small animals is crucial considering that 25% of European households own a pet. Antimicrobial treatment in dogs and cats is most commonly used against skin and wound infections, typically caused by *Staphylococcus pseudintermedius*, otitis externa caused by *P. aeruginosa*, and urinary tract infections (UTI) that can be caused by several bacteria including *E. coli* [14]. In 2009, the first case of human infection with methicillin-resistant *S. pseudintermedius* (MRSP) was reported [15]. Infections caused by MRSP (the main reason for antibiotic prescription in dogs) are challenging to eradicate, but MRSP is still considered a rare pathogen in humans, albeit with an increasing number of cases reported each year [16].

Emergence of AMR is often ascribed to overuse/misuse of antimicrobial drugs, as well as the pharmaceutical companies’ lack of success in meeting this challenge [17]. A contributing factor is the fact that development of antibiotics has a high risk profile due to single short-term prescriptions and restrictions on their use, and hence antibiotics often generate low returns [18]. In recent years, the AMR crisis has been addressed by several global organizations, attempting to raise global awareness [19]. Finally, a number of recent regulatory incentives for antibiotic drug development have been proposed as reviewed by Sinha [20].

### 1.1. Antimicrobial Resistance

Antimicrobial resistance may be intrinsic, adaptive or acquired. Intrinsic resistance implicates an ability of the pathogen to resist the antibacterial treatment due to inherent structural or functional properties, while adaptive resistance refers to an ability of the bacteria to adapt to non-lethal conditions by rapidly altering their transcriptomes in response to a stressful environment (e.g., selection pressure exerted by subinhibitory levels of antibiotics). Acquired resistance develops through acquisition of genes or as a result of a gene mutation, and it is the only form of resistance that can be transferred both horizontally and vertically [21]. Horizontal gene transfer implicates the exchange of genes between distinct species, while vertical gene transfer represents a transmission of DNA from the parent organism to its offspring [22]. There are several different main types of antibiotic resistance mechanisms: (i) Prevention of access of the antibiotic to its target by either reduced permeability of the membrane or by increased efflux [23]; (ii) Change in antibiotic target by mutations not affecting its functionality, but preventing interaction with the antibiotic [24]; (iii) Post-translational modification of the target [25]; (iv) Direct modification/degradation of antibiotics through enzyme-catalyzed transformations (e.g., hydrolysis of β-lactam antibiotics) [26].

### 1.2. Development of Antibiotics in Recent Years

For more than 70 years antibiotics have constituted a successful cure to life-threatening infectious diseases. Meanwhile bacteria evolved and became resistant, but antibiotic research was not highly prioritized by most of the pharmaceutical companies [27]. Today, the antibiotic pipeline is drying out, and efficacy of current antibiotics to combat MDR Gram-negative bacteria is rapidly declining. According to FDA, between 2012 and 2017 only nine antibiotics were approved, of which none was first-in-class against Gram-negative pathogens [28]. Notably, most newly approved antibiotics belong to known classes of antibiotics, and hence there is a high risk of rapid resistance development. For example, an MRSA strain resistant to linezolid was isolated only a year after its introduction to the market [29]. There is a steady state of antibiotics entering and leaving clinical trials [30]. In phase III clinical trials there are only eight drugs with broad-spectrum activity, while phase II trials comprise ten broad-spectrum drugs of which only one targets Gram-negative pathogens, whereas there is only one broad-spectrum antibiotic in phase I trial [28]. Additionally, in phase I–III there are six β-lactam/β-lactamase inhibitor combinations of known drugs that target Gram-negative bacteria. The lack of treatment options especially against MDR Gram-negative pathogens highlights the need for novel antimicrobial treatment strategies.

### 1.3. Antimicrobial Peptides (AMPs)

Antimicrobial peptides (AMPs), also often termed host-defense peptides (HDPs), have for the last two decades been considered as possible candidates for novel antibiotics due to their key role in the innate immune system in all multicellular organisms [31]. Thus, AMPs have been found not only in animals, but also in plants, fungi and even in bacteria. Today, all reported naturally occurring and synthetic AMPs are collected in databases, e.g., Data Repository of Antimicrobial Peptides (DRAMP), which contains more than 4500 sequences of antimicrobial peptides [32]. AMPs are typically divided into four groups based on their secondary structure [33]: (i) α-helical AMPs (e.g., the cathelicidins); (ii) β-sheet-containing AMPs, often with two or more disulfide bonds present (e.g., α- and, β-defensins); (iii) AMPs with a β-hairpin or loop conformation stabilized by the presence of a single disulfide bond and/or cyclisation of the peptide chain (e.g., thanatin); (iv) short AMPs with extended conformations (e.g., indolicidin).

Generally, AMPs contain 10–50 amino acids of which approximately half typically are hydrophobic, and most AMPs possess a net positive charge [34], although a few anionic AMPs are known [35]. Most AMPs are amphipathic, and this is believed to facilitate a selective electrostatic interaction with the negatively charged bacterial membranes, thereby causing perturbation of the lipid bilayer structure [36].

Despite increased attention to AMPs and the constant development of new biophysical techniques, the understanding of their mechanism of action at the molecular level remains to be elucidated in detail. The combination of cationic and hydrophobic residues promotes a strong electrostatic interaction with the negatively charged surface layer of bacteria that consist of lipids such as phosphatidylglycerol (PG), cardiolipin (CL) as well as lipopolysaccharides (LPS) in case of Gram-negative bacteria, or lipoteichoic acid (LTA) in case of Gram-positive bacteria. Additionally, hydrophobic interaction with bacterial envelopes induces folding of the AMPs that contributes to the antibacterial properties of these peptides. The high abundance of neutral phosphatidylcholine/cholesterol/sphingomyelin lipids confers a zwitterionic surface to mammalian cell membranes, which leads to a weaker attraction between cationic peptides and host cells, which is the origin of the cell selectivity observed for many AMPs. The bacterial membrane is considered as the main target for AMPs, since several studies have demonstrated that AMPs disrupt bacterial membrane integrity, thereby causing leakage of cell content followed by cell death [37]. A number of models of AMP disturbance of bacterial membranes have been reported, and these comprise: the carpet model [38]; aggregation [39]; molecular electroporation [40]; toroidal pore formation [41]; sinking raft [42]; barrel-stave model [43]; interfacial activity [44]; and lipocentric pore formation [45].

Although the bacterial membrane is the main AMP target, intracellular targets such as nucleic acid synthesis (e.g., buforin II), RNA synthesis (e.g., Bac5 and Bac7), protein synthesis (indolicidin), enzymatic activity (pyrrhocoricin), ATP efflux (histatins), or cell wall synthesis (nisin) have been reported [46].

### 1.4. AMPs as Therapeutic Agents

AMPs often display broad-spectrum antibacterial activity, including MDR pathogens, with a rapid onset of killing, and thus with relatively low risk of resistance development that make them desirable drug candidates. However, objective assessment of their therapeutic potential exposes a large difference between peptides claimed active in scientific articles and their realistic performance in clinical trials. Within the last three decades, despite numerous attempts, only four natural AMPs have found their way onto the market, none of them being linear peptides. Currently, no synthetic AMP has been approved by the FDA.

Several peptide antibiotics that have been introduced to the clinical use, e.g., bacitracin, polymyxin, gramicidin, and tyrothricin, are produced by *Bacillus* species [47]. Tyrothricin, the first AMP to be used clinically, consists of tyrocidine (main component) and gramicidin. Tyrocidine is a mixture of cyclic decapeptides that due to hemolytic properties exclusively are used as a topical agent [48]. Tyrocidine A and gramicidin S are broad-spectrum cyclodecapeptides that also only are applicable for topical use due to hemolytic side effects [49,50]. The cyclic lipopeptides polymyxin B and polymyxin E (also known as colistin) have been used for treatment of Gram-negative infections, and currently they constitute the last-line therapy for MDR Gram-negative infections [51]. Use of polymyxins is limited due to nephro- and neurotoxicity; however, via a prodrug formulation as the corresponding sulfomethylated compound acute toxicity is reduced. Bacitracin is a mixture of cyclic polypeptides used in combination with polymyxin and neomycin (Neosporin™) for topical treatment of eye and skin infections, however, bacitracin also exhibits nephrotoxicity [52,53]. Nisin is another natural (34-residue) AMP produced by *Lactococcus lactis*, which is effective against Gram-positive bacteria, in particular mastitis pathogens. Due to its low toxicity, nisin has been licensed in 48 countries for use as a food preservative especially in dairy products [54]. Finally, another cyclic lipopeptide, daptomycin, is utilized for treatment of infections caused by MDR Gram-positive pathogens [55].

In Table 1 and Table 2, selected AMPs currently undergoing clinical and preclinical trials as well as compounds recently failed during different stages are listed. Notably most AMPs failing preclinical studies during the last phase typically suffered from high susceptibility to degradation or from unexpected in vivo toxicity. Therefore, pharmaceutical companies have shifted focus from development of peptide drugs for systemic administration toward agents for topical use.

The majority of AMPs in current clinical development targets skin infections caused by Gram-positive bacteria. The magainin analog pexiganan is currently in phase III clinical trials for treatment of diabetic foot ulcers, though it was not approved by FDA in 1999 after completion of phase III. One of the most well-known host-defense peptides LL-37 is undergoing phase II for treatment of leg ulcer. After failing in phase III for possible treatment of catheter-related infections, the indolicidin analog omiganan is undergoing phase III for treatment of rosacea. However, there are several candidates exhibiting activity against Gram-positive and Gram-negative bacteria with alternative administration routes. For example, a cationic fragment of human lactoferricin hLF1-11 is aimed for intravenous treatment of bacterial infections in immunocompromised patients after stem cell transplants. Additionally, three AMPs, NVB-302, surotomycin and ramoplanin, are being developed for oral administration in order to treat infections caused by *Clostridium difficile*. The development of protegrin analog POL7080, the only AMP targeting Gram-negative infections, has been terminated during phase II after Roche discontinued their involvement. However, POL7080 was studied to investigate drug-drug interaction with amikacin and recently completed Phase I (January 2017) [56]. Polymyxin derivative SPR741, which acts as a potentiator in combination with antibiotics active against Gram-positive pathogens, has shown success in preclinical studies aimed at treating infections caused by Gram-negative bacteria, and it has entered Phase I clinical studies in January 2017 [57,58]. In addition, there are four AMPs in preclinical stage that exhibit activity against Gram-negative bacteria. The most recent advances in development of AMPs in clinical trials have been decribed more thoroughly in reviews by Greber & Dawgul [59], Kosikowska & Lesner [60] and Sierra et al. [61].

As an alternative to AMPs, rational design involving incorporation of non-standard amino acids has been explored as a strategy for obtaining compounds with AMP-like properties, where AMPs serve as starting pharmacophores of which length, backbone, side chains, hydrophobicity, charge and amphipathicity may be subjected to optimization. The resulting peptide mimics (or peptidomimetics as they will termed hereafter) often possess improved bioavailability and metabolic stability while retaining activity and selectivity profiles resembling those of AMPs. The term peptidomimetic usually refers to any sequence that is designed to mimic a peptide structure and/or function, but in which the backbone is not based on α-amino acid residues alone. To date, numerous strategies have been explored—from incorporation of a single unnatural amino acid residue to alternative backbone structures in order to discover potent peptidomimetic-based drug leads exhibiting high cell selectivity and improved bioavailability [99].

**Figure 1:**
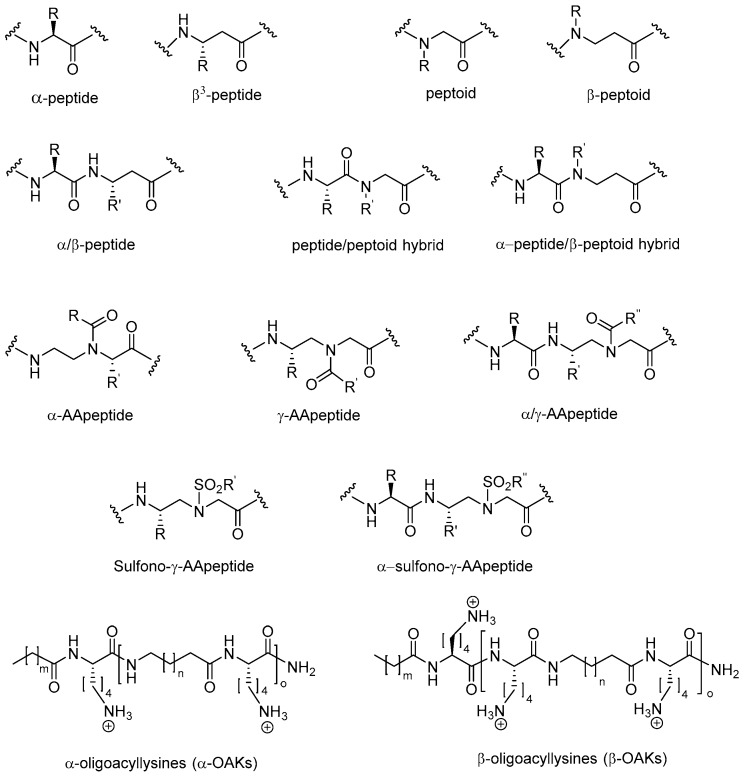
Basic structures of peptidomimetics discussed in the present review.

The most promising antimicrobial peptidomimetics that are readily optimized due to their modular structure include: *N*-alkylated glycines (peptoids), β-peptoids (*N*-alkylated β-alanine oligomers), β-peptides, α-peptide/β-peptoids, α/γ *N*-acylated *N*-aminoethylpeptides (AApeptides) and oligoacyllysines (OAKs). An advantage of such peptidomimetics (Figure 1) is that solid-phase synthesis allows for convenient preparation of large arrays of analogs by simple replacement/insertion of alternative residues by way of commercial and/or synthetic amino acid analogs. Also, these peptidomimetics possess enhanced in vivo stability and often exhibit lowered toxicity as compared to α-helical AMPs.

#### Resistance to AMPs and Peptidomimetics

Despite the interest in AMPs as antibiotic drugs only a few studies on resistance development have been reported. The general belief has been that resistance to AMPs is difficult to acquire since AMPs mainly target the bacterial membrane. However, studies have shown that bacteria can develop resistance to AMPs [100,101,102]. For example, Habets et al. demonstrated that experimentally evolved resistance to pexiganan provided *S. aureus* with cross-resistance to the defensin, human neutrophil peptide-1 [103]. A special concern is the possibility of bacteria becoming resistant to peptides of our own immune system after being exposed to unrelated therapeutic AMPs [104]. In their extensive review, Nawrocki et al. describe various mechanisms of AMP resistance for Gram-positive bacteria. Among all, Gram-positive bacteria have been a common source for AMPs, therefore there is a high likelyhood of resistance development in these pathogens that can be tranfrerred to non-producing strains [105]. Also, AMPs have been mostly targeting infections caused by Gram-positive bacteria, thereby increasing the risk of resistance development. Additionally, resistance mechanisms in Gram-negative bacteria evolved towards AMPs have been well described in a minireview by Gruenheid & Moual [106].

In one study it was shown that innate host-defense peptides are less efficient against AMP-resistant *S. aureus* thereby compromising the native immune response [107]. Hence, use of AMP drugs may prove to cause cross-resistance to human defensins and cathelicidins, but a better understanding of bacterial resistance to AMPs combined with new therapeutic strategies may overcome this problem [108].

For peptide/peptoid peptidomimetics it has been shown that resistant strains may be selected for by long-term continuous exposure in adaptive evolution experiments [109,110]. Hence, resistance is a key challenge also for peptidomimetics of AMPs when these are to be used more widespread as drugs.

### 1.5. Screening for Antimicrobial Activity and Cytotoxicity of Peptidomimetics

#### 1.5.1. Determination of Minimal Inhibitory Concentration

Determination of the minimal inhibitory concentration (MIC value) constitutes a key step in the evaluation of in vitro activity of potential new antimicrobial drug leads, and it is defined as the lowest concentration of an antimicrobial compound that inhibits the visible growth of a microorganism after overnight incubation (16–20 h). The resulting MIC value may be reported in either micromolar (µM) or µg/mL depending on the specific context. In medicinal chemistry the unit µM in often preferred as it allows for a direct comparison of the activity exerted by a concentration based on the number of molecules (typically measured in micromoles) rather than the amount in microgram it contains per volume. Micromolar concentrations are related to µg/mL as follows:

µg/mL = [(molecular weight including counterions)/1000] × µM

**Figure 2:**
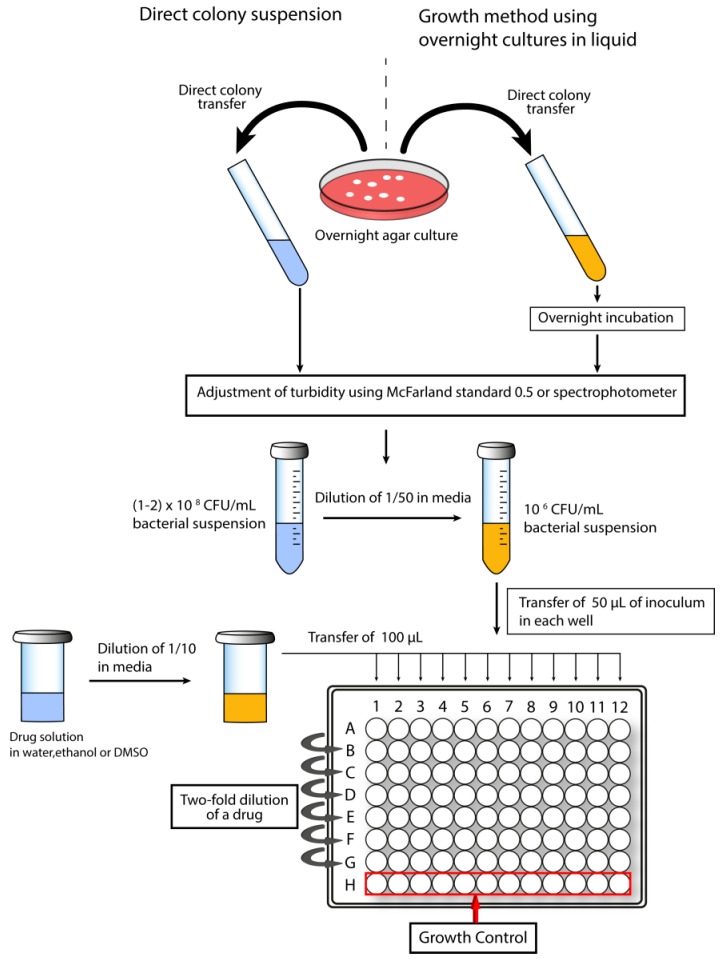
Determination of minimal inhibitory concentration by the broth microdilution method.

The Clinical and Laboratory Standard Institute (CLSI) and the European Committee on Antibiotic Susceptibility Testing (EUCAST) provide guidelines for MIC testing to ensure use of uniform and improved methodologies in various laboratories around the world [111]. Among a number of techniques, agar dilution and broth dilution are the most commonly used methods. For broth dilution, a two-fold dilution series of an antimicrobial drug in liquid growth media is inoculated with bacteria. Depending on the combined volume of test solution and bacterial inoculum, the method is defined as macrodilution (≥2 mL; performed in tubes) or microdilution (≤500 µL; performed in microtiter plates; Figure 2). A number of parameters may differ when a protocol for MIC determination is adapted in various laboratories.

Reproducibility of results relies on following the guidelines strictly. However, quite often a modified version of the recommended standard protocol is used in work reported in the literature. Change of parameters, e.g., type of media, incubation time, and concentration of bacterial inoculum, may give rise to misleading results. The ensuing inconsistency of literature data makes comparison of different antimicrobials quite difficult. Recently, some practical issues in how to carry out susceptibility testing of AMPs have attracted attention in the research community. The tendency of cationic AMPs to bind to polystyrene or glass surfaces was taken into consideration by Hancock et al. resulting in a modified CLSI-based protocol [112]. In this modified protocol, 0.02% acetic acid and 0.4% BSA (bovine serum albumin) are added to non-cation-adjusted medium (i.e., without addition of salts with divalent cations) in order to prevent binding of AMPs to the microtiter plates, and this may also improve solubility of peptides. This potential problem was investigated further by Gram and co-workers who showed that it was only critical when MICs are below 1 µM [113].

#### 1.5.2. Time-Kill Kinetics

**Figure 3:**
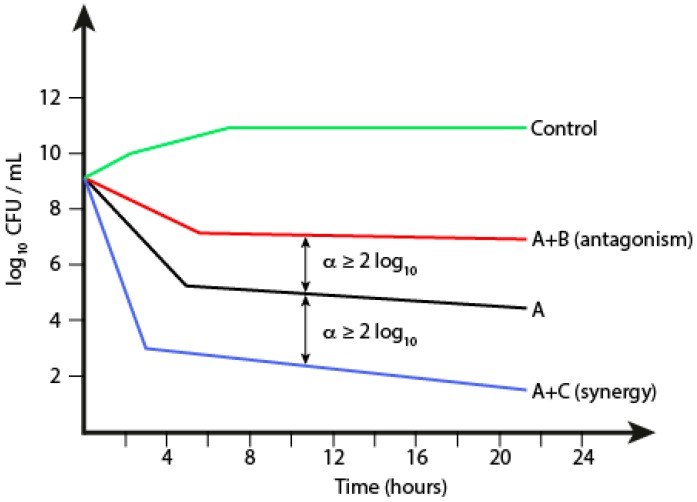
Schematic representation of a time-kill kinetics experiment.

The time-kill assay is the most appropriate method to assess pharmacodynamics of an antibacterial drug in vitro, as it reveals how fast bacteria are killed when exposed to either the MIC or slightly higher concentrations of an antimicrobial compound (Figure 3). At different time points the number of viable cells is counted by performing serial dilutions on an aliquot removed from the treated culture. Afterwards the concentration of surviving cells expressed as CFU/mL is plotted against time. In addition, the time-kill assay can be used to study drug interactions such as synergy and antagonism, as defined by 2 log_10_ decreased CFU/mL and 2 log_10_ increased CFU/mL, respectively, as compared to that found the most active single compound at a chosen time point. Cationic membrane-active AMPs are known to exhibit fast killing, while for AMPs with intracellular target typically an initial delay is seen [114].

#### 1.5.3. Synergistic Effects in Combination Therapy

**Figure 4:**
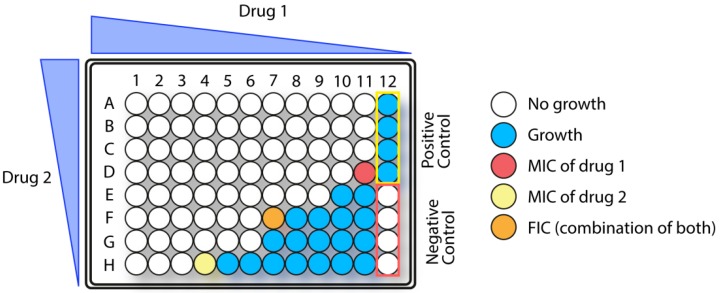
Example of a checkerboard testing of two drugs.

Antimicrobial combination therapy involves simultaneous use of two or more antibiotics, often with different mode of action, which may lead to synergy [115]. A synergistic effect implies that a combination of antibiotics show substantially higher activity than each antibiotic individually. Two widely used methods for testing of synergy between antimicrobial drugs are the checkerboard and time-kill methods. The time-kill method is used to compare the rate and efficiency of bacterial killing exerted by each drug alone and in combination. The checkerboard method (Figure 4) is often used for screening purposes, while the time-kill assay can provide a more detailed picture of a synergistic effect. The checkerboard method resembles the method for MIC determination, and provides information on which antibiotic combinations inhibit bacterial growth [116]. The latter method is usually performed by multiple dilutions of two different drugs in a microtiter plate that creates a checkerboard pattern, hence the name. Calculation of the fractional inhibitory concentration index (FICI) is used to analyze the results of the checkerboard assay by estimating the degree of synergistic effect. FICI is calculated as the sum of the individual fractional inhibitory concentrations (FICs) for each drug (where MIC A and MIC B denote the MIC of each drug alone, and MIC A_A+B_ and MIC B_A+B_ denote the concentrations of A and B in the drug combination):

FICI = (MIC A_A+B_/MIC A) + (MIC B_A+B_/MIC B)

The FIC for each drug is defined as the MIC of the combination divided by the MIC of the drug alone. Thus, the more efficient the synergy is, the lower the FICI will be. For a FIC index < 0.5, the combination of antibiotics has a synergistic effect, while a FIC index of 0.5–1.0 indicates that the effects of the drugs are additive. When FIC is between 1 and 4, the combination effect is indifferent, whereas a FIC > 4 indicate an antagonistic combination.

#### 1.5.4. Biofilm Eradication

**Figure 5:**
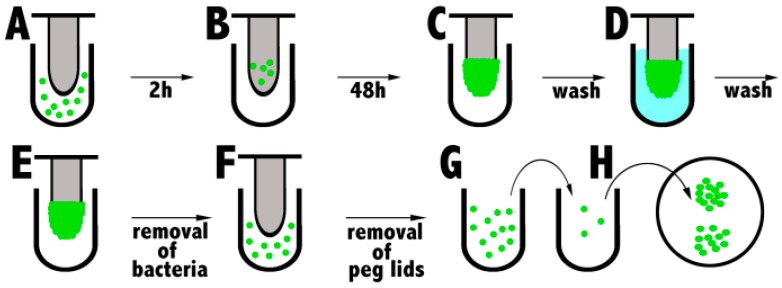
Diagram of experimental procedure for peg lid biofilm assay.

Bacterial growth within biofilms is associated with high tolerance to antibiotics and rapid development of resistance due to slow or incomplete killing. The key characteristic of biofilms is their ability to adhere to a surface, establishing a matrix of extracellular polymers. This feature allowed Ceri et al. to grow biofilms on peg lids [117]. A key step in the peg lid insertion method is the removal of pegs that allows for control measurement (e.g., microscopy), which cannot be performed by using direct cultivation in microtiter plates. In 2006 Harrison published the Calgary Biofilm Device (CBD) protocol that was slightly modified later in 2010 [118]. In brief, the peg lid is inserted into a 96-well microtiter plate containing bacterial inoculum in each well (A) (Figure 5). The peg lid with established biofilm is rinsed after incubation for 48 h (or more) to remove loosely adherent cells (C), and then it is inserted into another 96-well microtiter plate, containing serial dilutions of antibacterial drug (D). The peg lid is washed again (E), followed by staining with crystal violet or removal of surviving biofilm from the individual pegs (e.g., sonication, incubation in a growth medium) (F,G). Aliquots from each well are plated, incubated and CFU is counted to determine the minimal biofilm eradication concentration (MBEC) (H).

#### 1.5.5. Cytotoxic Activity

Cell selectivity, i.e., relative preference for killing bacteria over host cells, is a critical property of an antimicrobial drug. There are two commonly used ways to assess cellular toxicity of a compound. Testing for hemolytic activity (HA) is a standard inexpensive fast screening method by which the degree of lysis of red blood cells (RBCs) is measured after exposure of RBCs to the drug. This property indicates the propensity of test compounds to disrupt mammalian RBCs by measuring the amount of hemoglobin released from the lysed RBCs. HA is usually presented as an effective concentration of the compound resulting in 10% (HC_10_) or 50% (HC_50_) lysis of erythrocytes. HA is measured by incubation of serial compound dilutions in phosphate-buffered saline (PBS) with a suspension of freshly drawn red blood cells in PBS at 37 °C for a fixed time period (typically 1–8 h) in 96-well microtiter plates, where melittin and PBS often serve as positive and negative control, respectively [119]. Release of hemoglobin is measured on a spectrophotometer at 414 nm. Assessment of hemolysis is useful for screening purposes only, as a non-hemolytic compound may still be toxic toward other cell lines. However, it is a powerful tool that allows for a fast in vitro identification of highly toxic compounds. One of the disadvantages of this method is the often inconsistent choice of blood source. Due to absence of an internationally standardized protocol, research groups occasionally use blood of different origin e.g., human, rat, rabbit, sheep, horse etc., leading to incomparable results. Even when the choice is between different types of human blood, some issues must be considered in order to avoid any side interactions that can influence the final result. Therefore, type-0 blood is often considered the most appropriate option, since it lacks the saccharide antigens that can be found in types A and B. The noise level of the absorbance reader (~4%) also constitutes a disadvantage, because it restricts sensitivity of the method and complicates interpretation of data below 8% (2× noise level).

Determination of the effects of a compound on the viability of a panel of human cells is recommended as a standard protocol to provide a solid base for estimating its toxicity profile [120]. The viability assay is conducted by exposing cells grown in 96-well plates to a serial dilution of a drug in the medium followed by incubation under shaking at 37 °C. Tetrazolium dyes are used to stain healthy cells and to provide convenient conditions for spectrophotometric analysis. The MTT ((3-[4,5-dimethylthiazol-2-yl]-2,5-diphenyltetrazolium bromide) assay is one of the most common tools for detection of cytotoxicity or cell viability in vitro [121]. MTT is a water-soluble tetrazolium salt, which is converted into an insoluble purple formazan when exposed to metabolically functional mitochondria. Formazan cannot penetrate the cell membrane, and therefore it accumulates in healthy cells. The MTT assay is a highly sensitive method, however, insolubility of formazan in the cell medium may be a complication that can be solved by adding a solubilizing agent prior to measurement. An alternative tetrazolium dye, MTS (3-(4,5-dimethylthiazol-2-yl)-5-(3-carboxy-methoxyphenyl)-2-(4-sulfophenyl)-2*H*-tetrazolium), has been created to yield soluble formazan products, and when used the protocol is termed a ‘one-step’ MTT assay [122]. This method is successfully used to assess cytotoxicity of AMPs and peptidomimetics.

Other approaches for assessing the data quality in predictive toxicology have been described by Pohjala et al. [123] including the lactate dehydrogenase (LDH) assay, the water-soluble tetrazolium salt (WST-1) assay, and an assay measuring the intracellular level of ATP. Also LDH and gamma glutamyl transferase (GGT) release assays can be used for an in vitro prediction of drug-induced nephrotoxicity [124].

## 2. Peptidomimetics

The focus of this review is to highlight the developments in the last decade regarding the synthesis, discovery and potential applications of antimicrobial peptidomimetics (Figure 1) comprising the classes of α-peptoids (*N*-alkylated glycine oligomers), β-peptoids (*N*-alkylated β-alanine oligomers), β^3^-peptides, α/β^3^-peptides, α-peptide/β-peptoid hybrids, α/γ *N*-acylated *N*-aminoethylpeptides (AApeptides) and oligoacyllysines (OAKs). These classes of antimicrobial peptidomimetics are of particular interest owing to the convenient synthesis of the corresponding building blocks and their assembly by using standard solid-phase procedures. Other types of promising antimicrobial peptidomimetics not discussed in the present review comprise arylamide and phenylene ethynylene oligomers [125,126,127], small synthetic antimicrobial peptidomimetics (SMAMPs or SAMPs) [128,129,130], pyrazole, poly-lysine and histidine-derived ultra-short antimicrobial peptidomimetics [131,132,133], macrocyclic peptidomimetics [134], binaphthyl-based, functionalized oxazoles, biaryl amino acid templates in cyclic β-hairpin cationic antimicrobial peptidomimetics [135], thiazole peptidomimetics [136], 1,4-dihydropyridine cationic peptidomimetics [137], and peptidomimetics containing 3-aminobenzoic acid [138]. Several such small-molecule mimetics have been reviewed by Ghosh & Haldar [139]. For an authoritative review on general design and properties of peptidomimetics see Liskamp et al. [140].

### 2.1. Peptoids

**Figure 6:**
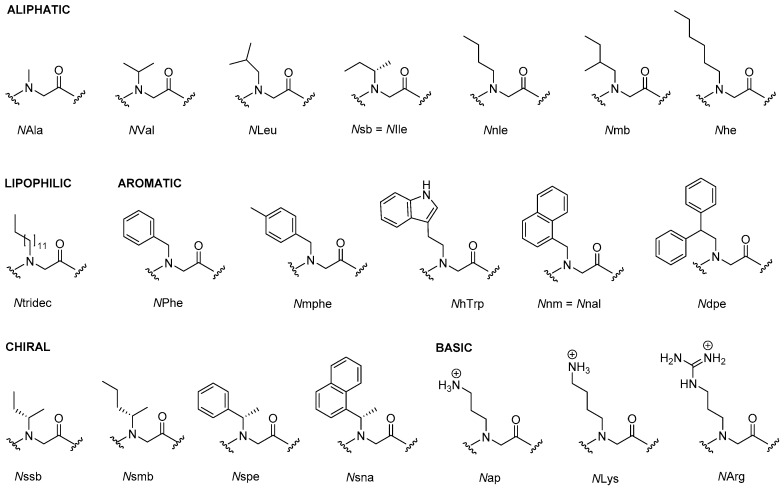
Structure of commonly used peptoid building blocks. *N*Ala: *N*-methylglycine; *N*Val: *N*-isopropylglycine; *N*Leu: *N*-isobutylglycine; *N*sb: *N*-(*sec*-butyl)glycine; *N*nle: *N*-(butyl)glycine; *N*mb: *N*-(2-methylbutyl)glycine; *N*he: *N*-hexylglycine; *N*tridec: *N*-tridecylglycine; *N*Phe: *N*-phenylmethylglycine; *Nm*phe: *N*-(4-methylphenyl)methylglycine; *N*hTrp: (*N*-(2-indolethyl)glycine; *N*nm: *N*-(naphtalen-1-ylmethyl)glycine; *N*dpe: diphenylethylglycine; *N*ssb: *N*-(*S*)-(*sec*-butyl)glycine; *N*smb: (*S*)-1-methylbutylglycine; *N*spe: *N*-(*S*)-(1-phenylethyl)glycine; *N*sna: (*S*)-(1-(naphthalen-1-yl)ethyl)glycine; *N*ap: *N*-(3-aminopropyl)glycine; *N*Lys: *N*-(4-aminobutyl)glycine; *N*Arg: *N*-(3-guanidinopropyl)glycine. Note: *N*Arg is obtained by reacting *N*ap with pyrazole-1-carboxamide on solid phase [161]. Adapted and expanded from Mojsoska et al. [162].

Peptoids are oligomers of *N*-substituted glycines and differ from α-peptides in that the side chains are attached to the backbone *N*^α^ position instead of at the C-α atom (Figure 6). Since peptoids often retain the biological activity of the parent peptide and are stable to proteases, they hold promise as therapeutics [141]. For recent reviews on peptoids see [142,143,144,145,146]. A historical account detailing the initial discovery and early developments in the peptoid field was published by Zuckerman [147]. Besides being promising antimicrobial agents, peptoids with activity against fungi [148], Candida biofilm [149], tuberculosis [150], the neglected tropical disease leishmaniasis [151], and cancer cells [152] have been reported. Peptoids have also found applications within supra- and macromolecular engineering [153], biomaterials [145], protein peptoid mutagenesis [154], and as fluorescent analogs for study of delivery to the cell nucleus [155], Huntington’s disease [156], positron emission tomography (PET) [157], glycopeptoids [158], DNA-binding peptoids [159], and carboxyalkyl peptoid PNAs [160].

#### 2.1.1. Synthesis

**Scheme 1:**
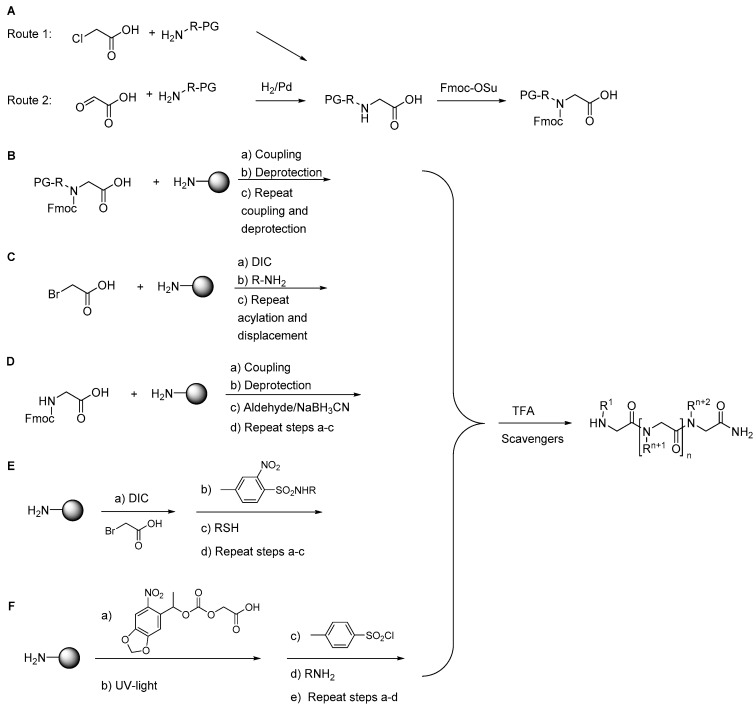
Overview of peptoid synthesis. Two different methods for preparing protected peptoid building blocks (**A**) for solid-phase synthesis (**B**); Submonomer approach developed by Zuckerman et al. (**C**); Submonomer reductive amination of Gly (**D**); Peptoid synthesis using *N*-substituted o-nitrobenzenesulfonamide derivatives (**E**); Photolithographic peptoid synthesis (**F**); Adapted from Simon et al. [163] and Gangloff et al. [153].

Peptoid synthesis is carried out by solid-phase synthesis (SPS) via the monomer or submonomer approaches. In the original monomer strategy [163] (Scheme 1A), a protected peptoid building block is synthesized by nucleophilic displacement of an alkyl halide by an appropriate amine (Route 1), or reductive amination (Route 2) followed by *N*-protection with Fmoc-OSu. The fully protected building block is then used in standard Fmoc-based SPS (Scheme 1B). However, the monomer strategy is laborious and time-consuming when several different building blocks are required. Shortly after, the submonomer approach was introduced by Zuckerman et al. [164], thereby avoiding the need for synthesis of protected building blocks (Scheme 1C). Here, peptoid synthesis is achieved entirely by solid-phase steps: bromoacetic acid is activated with *N*,*N*-diisopropylcarbodiimide (DIC) and then reacted with a resin-bound amine in DMF; subsequent nucleophilic displacement with a primary amine gives the desired peptoid residue. This activation/substitution cycle is repeated until the target sequence is obtained. Finally, the peptoid is cleaved from the resin by using TFA.

Three other less widely used submonomer strategies are known (Scheme 1D–F). A solid-phase submonomer strategy based on reductive alkylation of resin-bound Gly with the appropriate aldehyde to obtain the desired *N*-alkylated glycine derivative was reported by Tal-Gan et al. [165] (Scheme 1D). More recently, *N*-substituted *o*-nitrobenzenesulfonamide derivatives were used as alternative building blocks in the submonomer synthesis of peptoids as proposed by Vézina-Dawod et al. [166] (Scheme 1E). Finally, a four-step photolithographic synthesis of peptoids, using the [(α-methyl-2-nitropiperonyl)oxy]carbonyl (MeNPOC) group has been demonstrated by Li et al. [167] (Scheme 1F).

Zuckermann’s submonomer synthesis is most often performed on a Tenta Gel Rink amide resin using a two-step procedure. In the first step, acylation is typically performed by mixing the resin with bromoacetic acid (1.2 M; 20 equiv) in DMF and DIC (20 equiv) for 20 min. In the second step, nucleophilic S_N_2 displacement of the bromide is carried out with various primary amine solutions (1.0−2.0 M; 20–40 equiv) in DMF or *N*-methyl-2-pyrrolidone (NMP) during agitation for 90 min. Following synthesis, the product is cleaved from the resin with TFA/H_2_O/triisopropylsilane (95:2.5:2.5). Peptoid chains up to 50 residues can be synthesized in reasonable yield by using this approach [145]. Since 230 primary amines have been used in peptoid synthesis [142], and more than 1000 are commercially available this approach allows for the creation of a vast diversity. Besides bromoacetic acid, chloroacetic acid [168] and iodoacetic acid (via a Finkelstein reaction) [169] have been used. These are particular useful when heterocyclic aromatic amines are to be displaced [168] or for the chemoselective incorporation of functionalities into peptoids [169].

#### 2.1.2. Other Synthetic Developments

An approach enabling convenient synthesis of linear and cyclic peptoids that contain both arginine- and lysine-type residues within the same sequence has been reported. In this strategy pyrazole-1-carboxamide was used for on-resin guanidinylation of amines liberated upon *N*-Dde deprotection, while final lysine residues remained Boc-protected [161]. Furthermore, René et al. [170] described the synthesis of Fmoc-protected *N*-substituted glycine building blocks corresponding to a selection of functionalized natural amino acids like tyrosine, tryptophan, cysteine and serine. Finally, Caumes et al. [171] have described a gram-scale solution-phase synthesis applicable to β- and α,β-tetrapeptoids as well as α-peptoids. A strategy for the on-resin synthesis of antimicrobial macrocyclic peptides (3- to 13-mers) using the submonomer approach for cyclisation was described by Oddo et al. [172].

#### 2.1.3. Side Reactions

Not all primary amines are suitable for peptoid synthesis [142,173]. These include 2-methoxyethylamine and 2-phenylethylamine, which form *N*-substituted diketopiperazines; 4-(2-aminoethyl)morpholine and *N*^Im^-tritylhistamine, which have a nucleophilic group that also may react with the main-chain bromoacetyl group; 2,4,6-trimethoxybenzylamine and *p*-guanidino-substituted α-methylbenzylamine which is unstable during TFA cleavage. Peptides and peptoids are often acetylated at the N-terminus to improve biological activity; Kim et al. [174] reported that *N*-acetylated peptoids may spontaneously truncate under standard TFA cleavage conditions. They found that the problem could be circumvented by using a cleavage cocktail of TFA/CH_2_Cl_2_/H_2_O/TIS (50:42:5:3) for 1 h. Finally, it should be mentioned that HOBt and HOAt reduce the yield significantly when employed as additives in solid-phase submonomer synthesis [147].

#### 2.1.4. Antimicrobial Activity

**Figure 7:**
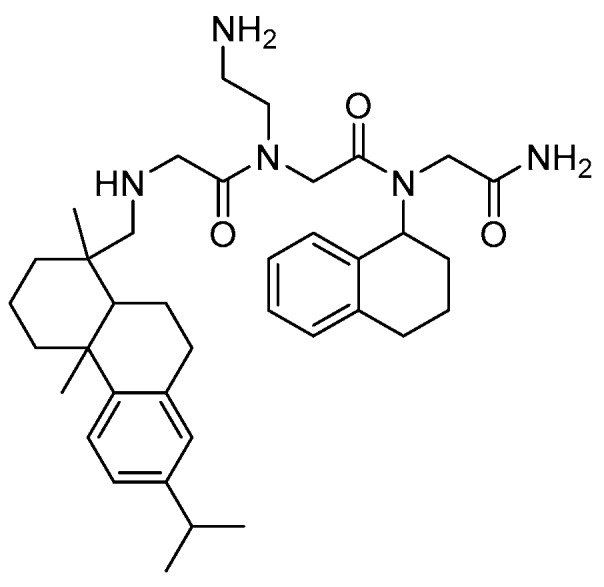
Structure of CHIR29498.

Antimicrobial peptoids were first reported by Goodson et al. [175,176] who identified a number of trimer peptoids from a combinatorial library of 845 members. Compound CHIR29498 (Figure 7) was the most active against a panel of resistant Gram-positive and Gram-negative bacteria with MICs ranging from 5 to 40 µM and displaying 40% hemolysis at 150 µM. Mice infected i.p. with 10^8^ CFU of *S. aureus* followed by i.p. injection of CHIR29498 (10 mg/kg) were protected five days after infection [175].

#### 2.1.5. Structure-Activity Studies of Peptoids

A structure-activity study of 22 cationic and amphipathic antimicrobial peptoids was reported by Mojsoska et al. [162]. Starting from known antimicrobial peptides (GN-2, -4 and -6), the authors described the transformation into peptoids, and investigated the effects of increasing hydrophobicity, single monomer substitution and variation of chain length. The most active compound H-*N*Lys-*N*hTrp-*N*Lys-*N*Lys-*N*hTrp-*N*hTrp-*N*Lys-*N*hTrp-*N*Phe-NH_2_ showed an MIC of 8, 16 and 2 µg/mL against MRSA, *E. coli* and *P. aeruginosa*, respectively, as well as an HC_50_ of 104 µg/mL. Two peptoids showed high cell selectivity and a bactericidal mode of action against *E. coli*. This was further investigated in a follow-up study which suggested that these peptoids act via a mechanism involving both membrane disruption and intracellular targets [177].

Chongsiriwatana et al. [178] reported a structure-activity study based on an array of 15 peptoids derived from H-(*N*Lys-*N*spe-*N*spe)_4_-NH_2_ and H-(*N*Lys-*N*ssb-*N*spe)_4_-NH_2_ by varying chirality, length, hydrophobicity, overall charge and amphipathicity. The authors observed: (i) the mechanism does not involve stereospecific interactions, which also has been shown for most AMPs; (ii) optimal hydrophobicity with highest antimicrobial potency was seen for 12-mers (while increased hydrophobicity conferred high hemolytic activity); (iii) helicity is required for hemolytic activity but not for antimicrobial activity; (iv) highly charged (≥+3), moderately hydrophobic and helical peptoids exhibit high cell selectivity. Ten of the 15 peptoids showed MICs against *B. subtilis* and *E. coli* similar to those of melittin and pexiganan.

A structure-activity study involving 44 peptoids was reported by Bolt et al. [179]. The compounds were six to twelve residues in length with a net charge ranging from +2 to +4. Generally, the peptoids were considerably more active toward the Gram-positive bacteria *S. aureus* and *S. epidermidis* than against the Gram-negative bacteria tested. Several peptoids displayed activity (Table 3) against *E. coli* (MIC of 6 μM for five compounds) and *P. aeruginosa* (MIC of 13 μM for three compounds). However, these compounds were also toxic against two mammalian cell lines HaCaT and HepG2 (EC_50_ below 50 µM). In agreement with previous studies, the authors observed that antimicrobial activity was correlated with length: 12 residues > 9 residues > 6 residues. Furthermore, fluorine-substituted analogs displayed increased antimicrobial activity, and achiral sequences were more active and less hemolytic than peptoids with a higher content of chiral monomers.

Using a number of in silico, in vitro, and in vivo techniques, Czyzewski et al. [180] reported a QSAR model based on 27 peptoid sequences, which accurately correlated structure of antimicrobial peptoids with their antimicrobial activity. The most active compound was H-(*N*Lys-*N*spe-*N*spe)_4_-NH_2_ (discussed above), which exhibited MICs ≤8 μg/mL for 19 out of the 20 MDR strains tested, and it was more active than the AMPs MSI-78 and MX-226. In a murine model with invasive *S. aureus*, the peptoid was able to reduce bacterial counts two log orders in the peritoneum at a concentration of 4 mg/kg without causing any medium-term toxicity.

Bang et al. reported the conversion of a Trp-rich peptide, H-KLWKKWKKWLK-NH_2_, into the corresponding protease-stable peptoid without loss of potency or increase in hemolytic properties [181]. Interestingly, the killing kinetics of the Trp-rich peptide and the peptoid analog were compared at 8 μM (1 or 2 × MIC) against *E. coli* and *S. aureus*, and it was found that the bactericidal rate of the peptoid (60 min) was slower than that of the peptide (30 min), indicating different killing mechanisms.

Synergy between peptides and peptoids against Gram-negative bacteria has been reported by Chongsiriwatana et al. [182] who studied combinations of seven peptoids and the AMPs pexiganan and melittin. Against *E. coli*, 7 of 36 combinations (e.g., H-*N*spe-*N*spe-[*N*Lys-*N*spe-*N*spe]_5_-NH_2_ and pexiganan) yielded FICIs ≤ 0.25, indicating highly synergistic interactions with at least an 8-fold decrease in the MIC of each compound in the presence of the other. The HC_50_ of the most active combinations ranged from 8 µM to >200 µM. The trend was that relatively hydrophilic oligomers exhibited synergism with hydrophobic oligomers, and it was suggested that synergy was due to distinct but complementary mechanisms.

Barron et al. [183] investigated the antimicrobial activity of H-(*N*Lys-*N*spe-*N*spe)_n_-NH_2_ (n = 1–4) and lipidated analogs carrying alkyl chains of 5, 10 or 13 carbons. The most active compound was H-*N*tridec-*N*Lys-*N*spe-*N*spe-*N*Lys-NH_2_ which exhibited MICs of 10.8 µg/mL against *P. aeruginosa* and *K. pneumonia* and 5.4 µg/mL against MRSA. The hemolytic activity (HC_50_) was 224 µg/mL. The peptoids H-(*N*Lys-*N*spe-*N*spe)_4_-NH_2_ and H-*N*tridec-*N*Lys-*N*spe-*N*spe-*N*Lys-NH_2_ were also shown to reduce the viability of *P. aeruginosa* (PA 14) biofilms at their MICs (12.5 µM and 12.5–25 µM, respectively), whereas antibiotics, such as kanamycin, showed comparable results at much higher concentrations [184]. Guanidinylation and tail effects in cationic antimicrobial trimeric lipopeptoids were studied by Findlay et al. [185] who synthesized 19 compounds consisting of combinations of *N*hArg, *N*Lys, Gly and a fatty acid or fluorinated fatty acid (9–20 carbon atoms) at the N-terminus. Most peptoids exhibited moderate activity against Gram-positive bacteria (MICs of 8–64 µg/mL) and weak activity against Gram-negative strains (MICs of 16–512 µg/mL). As expected, these lipopeptoids were also very hemolytic.

#### 2.1.6. Libraries

**Figure 8:**
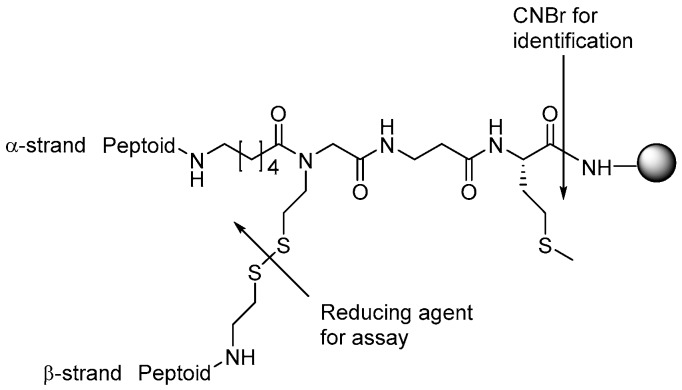
General structure of the Peptoid Library Agar Diffusion (PLAD) system. The identical peptoid sequences are cleaved separately: (i) with a reducing agent (e.g., a thiol) for screening of antimicrobial activity, and (ii) with CNBr to identify the active compound by mass spectrometry.

Lipophilic antimicrobial peptoids have been identified by testing of peptoids in a so-called peptoid library agar diffusion assay (PLAD) [186]. Resin beads containing two identical peptoid strands (α and β) are imbedded into soft agar together with the test microorganism (Figure 8). Overnight incubation with a reducing agent, e.g., β-mercaptoethanol, releases the β-strand peptoid. The other strand is cleaved with cyanogen bromide at the C-terminal methionine. The product is then analyzed by mass spectrometry to identify the active peptoid. In a follow-up study, the authors identified a peptoid which exhibited MICs of 25 μg/mL and 6.3 μg/mL against *A. baumannii* and *E. faecalis*, respectively, while it showed 30% hemolysis at 100 µg/mL [187]. The PLAD strategy has also been applied for identification of a peptoid with antifungal activity against *Cryptococcus neoformans* [148].

#### 2.1.7. Cyclic Peptoids

Huang et al. [188] investigated the effect of head-to-tail cyclisation on the antimicrobial activity of short peptoid sequences that contain cationic and hydrophobic side chains. Fifteen cyclic peptoids (6–10 residues), were compared to the corresponding acetylated linear peptoids. It was found that cyclisation and increasing length generally improved antimicrobial activity. The most pronounced example was Ac(*N*ap-*N*Phe)_5_ and c(*N*ap-*N*Phe)_5_, which against *B. subtilis*, *E. coli* and *S. aureus* showed MICs of 15.6 vs. 0.5 µg/mL; 31.3 vs. 7.8 µg/mL; and 62.5 vs. 7.8 µg/mL, respectively. The HC_50_ was not affected by chain length or cyclisation (>250 µg/mL for all compounds). In a follow-up study, the authors reported on 26 cyclic peptoids based on pentamer and hexamer scaffolds, containing cationic aminopropyl groups and a number of hydrophobic residues. Ten cyclic peptoids showed moderate MICs ranging between 15.6–250 µg/mL against clinical isolates of MRSA, while nine peptoids were very active (MICs ≤ 3.9 µg/mL). These peptoids target and damage the MRSA cytoplasmic membrane through formation of pores as demonstrated by electron microscopy experiments [189]. The same research group also studied the membrane interactions of linear and cyclic versions of the antimicrobial peptoids (*N*ap-Ndp)_3_ (Table 3) and (*N*ap-Nnm)_3_ by using scanning electron microscopy on bacterial cells, and high-resolution X-ray scattering and epifluorescence microscopy on Langmuir layers [190]. They found that cyclisation increases the membrane activity of antimicrobial peptoid oligomers, and suggested this to be related to the reduced conformational flexibility of the cyclic peptoids.

### 2.2. Peptides Displaying Substitution(s) with Peptoid Residues and Peptide-Peptoid Hybrids

The Shin group. has been a major contributor to the exploration of the effects of introducing one or more α-peptoid residues into α-helical peptides [191,192,193,194]. A key aim in these investigations was to improve the pharmacological profiles of hemolytic AMPs towards lower toxicicity while retaining antibacterial activity [191,192,193,194]. Similar to Pro, a peptoid residue constitutes a potential inducer of a β-turn and possess an ability to break an α-helix, since peptoid residues lack the amide proton required for stabilization via intramolecular hydrogen bonds. Typically, the insertion point of peptoid residue(s) is in the middle region of the sequence, thereby partially disrupting the α-helical conformation, which often gives rise to a change in mode of action from membrane permeabilization toward bacteria penetration to reach putative intracellular target(s) [192,193,194,195]. Likewise, replacement of Pro residues in Trp/Pro-rich AMPs appears to constitute a promising approach for development of cell-selective AMPs with intracellular mechanisms [196]. In most cases the modification with α-peptoid residues confers broad-spectrum antibacterial activity against common Gram-negative and Gram-positive pathogens [191,192,193,194,195,196,197,198]. With few exceptions preparation involves solid-phase peptide synthesis (SPPS) where Fmoc-protected peptoid building blocks are used at the appropriate positions.

#### 2.2.1. AMPs Displaying Substitution(s) with Peptoid Residues

Analogs of the plant-derived 20-residue 1b-AMP1, containing two disulfide bonds, were designed as readily synthesized linear peptides lacking the N-terminal Glu as well as the four Cys residues, but displaying a D-Pro or peptoid residue (*N*Ala or *N*Lys) instead of L-Pro in the central position of the sequence (WGRRGWXGRRYVRW-NH_2_, X = Pro or peptoid) [199]. All analogs exhibited approx. 4-fold enhanced activity (MICs in the range 2–16 µM) against both Gram-negative (*E. coli*, *P. aeruginosa*, and *S. typhimurium*) and Gram-positive (*S. epidermidis* and *S. aureus*) bacteria. Circular dichroism (CD) spectroscopy inferred that the modifications with peptoid residues induced a β-turn conformation in the presence of bacterial membrane-mimicking dodecyl phosphocholine (DPC) micelles. The analogs had weak ability to induce fluorescent calcein leakage from negatively charged liposomes that mimic bacterial membranes. Moreover, the analogs induced significant depolarization of the membrane potential in intact *S. aureus*, while the original 1b-AMP1 showed no effect in this assay. Collectively, these results suggest a mode of action involving formation of transient pores rather than membrane disruption [199].

Piscidin 1 from a hybrid of striped bass is an antibacterial and cytotoxic α-helical peptide (22 residues) for which analogs with peptoid residues (FFHHIFRXIVHVGKTIHRLVTG, X = P, *N*Ala, *N*Leu or *N*Lys) were designed [193,197]. The hydrophobicity of the *N*Ala- and *N*Leu-substituted analogs was higher than that of the *N*Lys-containing analog, but all peptides retained high antibacterial activity (MICs of 1–8 µM) against a panel of Gram-negative and Gram-positive bacteria, and were devoid of hemolytic activity at a concentration of 100 μM, whereas the original AMP was hemolytic at 3 µM.

The tertiary structures of the Pro- and *N*Lys-containing analogs (i.e., Pis-1[PG] and Pis-1[NkG], respectively) in the presence of DPC micelles were determined by NMR spectroscopy. While Pis-1[PG] had a rigid bent structure at the central Pro, Pis-1[NkG] was found to exist as two conformers with a flexible hinge structure at *N*Lys [193]. Confocal laser-scanning microscopy revealed that all fluorophore-labelled peptoid-containing analogs penetrated the bacterial membrane and accumulated in the cytoplasm, whereas Pis-1[PG] localized to the surface and displayed extensive membrane depolarization of *S. aureus* [193,197]. The different conformations induced by Pro and Gly replacement by a peptoid residue may account for the distinct killing mechanisms of the resulting analogs. Thus, introduction of an *N*Lys residue provided simultaneous conformational flexibility and increased cationicity in the hinge region, which facilitated penetration of the bacterial cell membrane and conferred the highest bacterial cell selectivity to Pis-1[NkG] [193,197]. Moreover, the most hydrophobic *N*Leu-substituted analog exhibited inhibition of LPS-induced nitric oxide production at a concentration of 2 µM [197].

Papiliocin is a 37-residue AMP from larvae of the butterfly *Papilio xuthus*, while magainin 2 is a 23-residue AMP isolated from the skin of the African clawed frog *Xenopus laevis*. Papiliocin−magainin 2 hybrid AMPs (18 residues: RWKIFKKIXKFLHSAKKF-NH_2_, X = P or *N*Lys) were constructed by linking the N-terminus (residues 1−8) of papiliocin and the N-terminus (residues 4−12) of magainin 2 via a central hinge region induced by either a Pro or an *N*Lys residue (designated PapMA and PepMA-k, respectively) [198]. PapMA had high antimicrobial activity but was cytotoxic to mammalian cells, whereas PapMA-k retained broad-spectrum high antimicrobial activity against a panel of Gram-negative and Gram-positive species (MICs in the range 2–16 µM), but was less hemolytic and cytotoxic (IC_50_ ~ 40 µM toward RAW264.7 and murine fibroblasts (NIH 3T3 cells)) than PapMA (IC_50_ ~ 10 µM). In addition, MICs in the range 2–8 µM were found against clinical isolates of MDR *P. aeruginosa*, *E. coli*, *A. baumannii*, MRSA, and *S. typhimurium*. NMR experiments revealed that the structures of PapMA and PapMA-k were consistent with *N*- and C-terminal helices connected via a hinge residue, which for *N*Lys gave rise to a dynamic equilibrium between *cis* and *trans* conformers. Fluorescent dye leakage experiments with liposomes mimicking bacterial membranes as wells as confocal microscopy of fluorescent analogs interacting with *E. coli* indicated that the mechanism of PapMA is membrane permeabilization, whereas PapMA-k is a bacteria-penetrating peptide with a putative intracellular target. Thus, the flexible structure as well as the positively charged *N*Lys residue appear to play important roles in the bacterial cell selectivity of PapMA-k. By using lipopolysaccharide-stimulated RAW264.7 macrophages, both hybrids were found to display anti-inflammatory activities, e.g., inhibition of production of nitric oxide and expression of pro-inflammatory cytokines at concentrations of 5–10 µM [198].

Gobbo et al. have investigated how replacement of Arg and/or Leu residues with an analogous peptoid residue in the Pro-rich insect AMP apidaecin Ib (GNNRPVYIPQPRPPHPRL) affected the properties [200]. Generally, introduction of *N*Arg residues resulted in reduced antimicrobial activity, however, modification in positions 4 or 12 led to non-hemolytic analogs (at least up to 300 µM) with only ~2-fold higher MICs (16–32 µM) against several Gram-negative bacteria. Peptoid substitution in the C-terminal Arg-Leu positions prevented the rapid enzymatic degradation seen for native apidaecin 1b that produced inactive truncated analogs. Unfortunately, these peptoid-containing analogs lacked antibacterial activity; similarly, a tris-*N*Arg-substituted analog lacked activity. Although peptoid-peptide hybrids are more resistant to proteolysis and devoid of any significant cytotoxic activity, shifting of the Arg → *N*Arg replacement from the N-terminus towards the *C*-terminus progressively reduced the antibacterial activity of the analogs. Incubation experiments with *E. coli* cells infer that loss of antibacterial activity of apidaecin diplaying a C-terminal 17-*N*Arg residue is a consequence of its inability to translocate into bacterial cells, supporting the involvement of a membrane peptide transporter (e.g., SbmA) [200]. SPPS of peptoid-peptide hybrids having an *N*Arg residue was found to be facilitated by submonomer assembly of the *N*-protected aminoalkyl glycine residue, followed by completing the sequence with an on-resin guanidinylation to give the final peptoid-peptide hybrid [200].

Melittin is a highly cytotoxic AMP (IC_50_ of 2 and 4 µM against human cervical carcinoma (HeLa) cells and NIH 3T3 cells, respectively) with potent broad-spectrum antimicrobial activity (MICs of 0.5–4 µM). In order to reduce cytotoxicity an analog (ME-w; GIGAVLKVLTTGLPALIS-*N*hTrp-IKRKRQQ-NH_2_) displaying a homo-Trp peptoid residue (*N*hTrp) was designed [191]. ME-w exhibited similar antimicrobial activity as melittin, but accompanied by lower hemolytic properties and reduced cytotoxicity against HeLa and NIH-3T3 cells (IC_50_ of 35 and 56 µM, respectively). Tryptophan fluorescence and CD spectroscopy revealed that the *N*hTrp-substituted analog had a much lower tendency to assemble into helical tetramers, which may explain the lower cytotoxicity [191].

Melittin contains a leucine zipper motif, consisting of every seventh amino acid (being Leu or Ile) that are aligned in a similar position in a helical wheel projection. In an attempt to generate cell-selective analogs, the residues in this zipper sequence were replaced by either *N*Ala, *N*Leu, *N*Phe, or *N*Lys (to give Me-a, Me-l, Me-f or Me-k) [195]. CD spectroscopy indicated that such substitutions disrupt the α-helical structure in the presence of zwitterionic phospholipid vesicles. Also, Me-l, Me-f and Me-k proved incapable of self-association in an aqueous environment with high ionic strength, which may be correlated to their non-hemolytic behavior even at 100 µM, whereas native melittin is hemolytic already below 1 µM. Likewise, when tested towards HeLa and NIH 3T3 cells melittin inhibited growth at ~10 μM, while ME-a, ME-l, and ME-k did not exhibit any cytotoxicity even at 100 μM. Broad-spectrum antibacterial activity against Gram-negative and Gram-positive species was observed for ME-f (MICs of 1–8 µM) and ME-k (MICs of 2–4 µM) corresponding to a ~2-fold lower potency as compared to that of native melittin. The peptoid-containing analogs were shown to possess little or no ability to induce membrane disruption or membrane depolarization. Substitution of the leucine zipper motif in melittin with peptoid *N*Leu or *N*Lys residues confers increased selectivity for bacterial cells by impairing self-association, and this modification changes the mode of antibacterial action from membrane disruption to a putative intracellular mechanism, and thus analogs displaying three *N*Leu or *N*Lys residues appear to be therapeutically useful lead compounds [195].

In order to convert the non-cell-selective Leu/Lys-rich model peptide KLW into an AMP with high preference for killing bacterial cells its two Phe residues were replaced by *N*Phe moieties to give the 18-mer KLW-f (KWKKLLKKfLKLfKKLLK-NH_2_; f = *N*Phe) [192]. In contrast to the distinctly α-helical KLW, the CD spectrum of peptoid-containing KLW-f inferred a disordered structure due to the helix-breaking properties of the peptoid residues inserted in these middle positions. KLW-f exhibited high antimicrobial activity (MICs of 0.5–2.0 μM) against both Gram-negative (e.g., *E. coli* and *P. aeruginosa*) and Gram-positive (e.g., *S. aureus*) bacteria. At a concentration of 100 µM, KLW-f was devoid of hemolytic properties and cytotoxicity towards HeLa and NIH 3T3 cells. In addition, KLW-f caused no or little dye leakage from bacterial membrane-mimicking vesicles, indicating that its mode of action does not rely on permeabilization or disruption of bacterial membranes. Furthermore, KLW-f showed a significant inhibition of LPS-induced NO production from mouse macrophages (RAW264.7 cells) at 10 μg/mL [192]. These findings suggest that KLW-f may constitute a potential lead compound in the development of antisepsis therapeutics and/or antimicrobial agents.

Similarly, double introduction of L-Pro, D-Pro, D-Leu or *N*Leu residues into the hydrophobic face of the α-helical non-cell-selective antimicrobial peptide L_8_K_9_W_1_ (KWKKLLKKXLKLXKKLLK-NH_2_; X = Leu), designed to possess a perfectly amphipathic structure, had significant effects on conformation, cell selectivity and mechanism of action [194]. The resulting analogs lacked α-helical structure and displayed increased selectivity toward bacterial cells; in particular, L_8_K_9_W_1_-D-Pro (X = D-Pro in the above sequence) and L_8_K_9_W_1_-*N*Leu (X = *N*Leu) had potent broad-spectrum antibacterial activity (MICs of 0.5–2 μM; including MRSA and MDR *P. aeruginosa*), but were non-hemolytic even at 100 µM. Interestingly, at 5- to 10-fold higher concentrations than their MIC the analogs containing an L-Pro, D-Pro or *N*Leu residue interacted weakly with negatively charged phospholipid vesicles that mimics bacterial membranes and caused weak depolarization of *S. aureus* membranes [194]. Thus, L_8_K_9_W_1_-l-Pro, L_8_K_9_W1-d-Pro and L_8_K_9_W_1_-*N*Leu may be promising candidates for novel therapeutic agents due to their high cell selectivity and activity against MDR strains.

In addition, three hemolytic non-helical Trp/Pro-rich AMPs were examined with respect to the effect of Pro → *N*Lys substitution on their cell selectivity and mode of action. All Pro residues in tritrpticin (TP: VRRFPWWWPFLRR-NH_2_), symmetric TP analog (STP: KKFPWWWPFKK-NH_2_) and indolicidin (IN; ILPWKWPWWPWRR-NH_2_) were simultaneously replaced with *N*Lys residues to give the analogs TPk, STPk and Ink [196]. The *N*Lys-substituted peptides (i.e., TPk, STPk and Ink) exhibited broad-spectrum antibacterial activity (MICs in the range 0.5–8 µM) against *E. coli*, *P. aeruginosa* and *S. aureus* including MDR strains, and displayed no hemolytic activity at 200 µM corresponding to at least 2- to 8-fold higher cell selectivity than the parent Pro-containing AMPs. In contrast to the original Pro-containing AMPs the *N*Lys-substituted peptides induced only weak depolarization of the membranes of intact *S. aureus* at their MIC. This suggest that also for non-helical Pro-rich AMPs incorporation of *N*Lys residues confers a mode of action that most likely involves interference with essential intracellular processes [196].

Table 4 shows the activity profiles for some of the most active AMPs displaying peptoid residues.

Recently, an array of AMPs, for which indications for intracellular target had been reported, was examined for their ability to act as a translocation vehicle for an antisense peptide nucleic acid (PNA) targeting fatty acid synthesis [201]. While TPk lacked capability of bacterial delivery of PNA, the antisense PNA oligomer conjugate with KLW-L9, 13-a (KWKKLLKK-*N*Ala-LKL-*N*Ala-KKLLK-NH_2_) displayed moderately enhanced activity as compared to the AMP itself independently of the expression of the SbmA peptide transporter [201].

#### 2.2.2. Peptide-Peptoid Hybrids

By integrating chemometrics and cheminformatics methods with in vitro susceptibility test a number of antibacterial peptide-peptoid hybrids were identified [202]. Descriptors for a panel of natural amino acids (e.g., Trp, Leu, Lys and Arg) and peptoid residues abundant in AMPs and antibacterial peptoids, respectively, were derived by principal component analysis of constitutional, topological, geometrical, and physicochemical properties. These were then used to model the correlation between structural features and antibacterial activity of known 9-mer AMPs. High-throughput virtual screening of a combinatorial library based on ten natural amino acids and eight peptoid residues allowed selection of potential antibacterial compounds, for which the potency against *S. aureus* and *P. aeruginosa* was determined in vitro. Two 8-mer peptide-peptoid hybrids (IK-*Ns*sb-*N*Lys-VRK-*N*ssb-NH_2_ and *N*Lys-*N*Lys-W-*N*smb-IKRW-NH_2_) were found to possess antibacterial activity with MIC values in the range 3–12 μg/mL against the two strains. Molecular dynamics simulations revealed that IK-*N*ssb-*N*Lys-VRK-*N*ssb-NH_2_ folds into a typical amphipathic helix that promotes membrane disruption, while *N*Lys-*N*Lys-W-*N*smb-IKRW-NH_2_ was devoid of secondary structure [202].

An array of twenty lysine-peptoid hybrids (5–10 residues), based on the hydrophobic *N*nal-Nmphe-*N*nal-*N*nle-NH_2_ hemolytic lead compound, was designed by incorporating a varying number of cationic Lys residues (1–6) distributed along the sequence in several display modes in order to explore the SARs within this subclass of peptidomimetics [203]. These hybrids were found to exhibit antimicrobial activity against clinically important bacteria including *E. faecium* (VRE strain), *S. aureus* (e.g., MRSA), and *P. aeruginosa* (ATCC 27853), whereas they were inactive against *K. pneumoniae* [204]. The general trends were that longer compounds displaying four or more lysines were most active, and that these were most potent against Gram-positive species. Several peptidomimetics displaying high activity against *S. aureus* strains and VRE (MICs 1.6–6.25 µM) were identified, with *N*nal-*N*mphe-*N*nal-*N*nle-KKKKK-NH_2_ (LP5) being the most active. The two most potent low-hemolytic compounds toward *P. aeruginosa* were KKKK-*N*nal-*N*mphe-*N*nal-*N*nle-NH_2_ and KKKKK-*N*nal-*N*mphe-*N*nal-*N*nle-NH_2_ [204]. In a recent study LP5 was shown to retain activity under settings resembling host physiological conditions with respect to salinity, Mg^2+^ concentration and pH. However, in the presence of serum, LP5 lost activity. Moreover, at high salinity or lowered pH, the peptidomimetic exhibited reduced activity. Interestingly, exposure of *S. aureus* to subinhibitory concentrations of LP5 affected the expression of the major virulence factors of *S. aureus* [205]. Table 5 shows the activity profiles for some of the most active peptide-peptoid hybrids.

### 2.3. β-Peptoids

**Figure 9:**
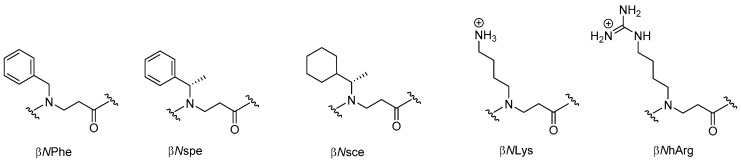
Structure of commonly used β-peptoid building blocks. β*N*Phe: *N*-(1-phenylethyl)-β-alanine; β*N*spe: *N*-(*S*)-(1-phenylethyl)-β-alanine; β*N*sce: *N*-(*S*)-(1-cyclohexylethyl)-β-alanine; β*N*Lys: *N*-(4-aminobutyl)-β-alanine; β*N*hArg: *N*-(3-guanidinobutyl)-β-alanine.

Conceptually, β-peptoids may be considered as the homologues of ordinary α-peptoids in the same way as β^3^-peptides corresponds to backbone methylene-elongated α-peptides. However, despite its apparent simplicity the subclass of *N*-alkylated β-alanine oligomers (typical monomers are depicted in Figure 9) was for long only sporadically explored [206]. Whether β-peptoids might have an advantage as compared to α-peptoids or other foldamers remains to be elucidated, but as recently reviewed by Olsen [207] the prospect of gaining more detailed knowledge on the factors that control their folding propensity may clearly extend the scope of these peptidomimetics further.

From a synthesis point of view a major obstacle was the encountered difficulties in developing fast and efficient solid-phase routes to longer β-peptoid oligomers, since the obvious submonomer approach involving on-resin aza-Michael conjugate addition of a primary amine to a resin-bound acrylamide intermediate proved to be hampered by long reaction times [208,209]. For β-peptoid oligomers displaying α-chiral side chains even the monomer-based strategy using Fmoc-protected building blocks proved challenging [210], while achiral oligomers readily could be obtained via microwave (MW)-assisted SPS under conditions similar to those applied in traditional SPPS [211]. Also, a block synthesis protocol has been reported [212], whereas cyclic β-peptoids have been assembled via a solution-phase method [213].

Although only two reports on antibacterial β-peptoids have appeared so far [211,212] a comparative study comprising several subclasses of peptidomimetics showed that the β-peptoid backbone conferred similar antimicrobial activity to the resulting oligomer as other templates (e.g., α-peptoid or β^3^-peptide) when displaying an alternating sequence of cationic/hydrophobic side chains [211]).

#### 2.3.1. Synthesis of β-Peptoid Oligomers

**Scheme 2:**
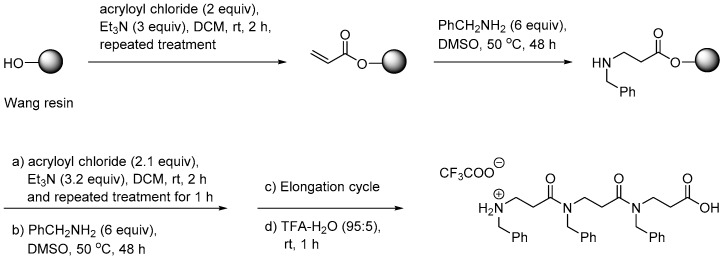
SPS of achiral β-peptoids via the submonomer aza-Michael method [208].

The first report on solid-phase synthesis (SPS) of β-peptoid oligomers concerned assembly of a combinatorial library of trimers starting from an acryloylated Wang resin. Elongation was performed via a submonomer approach involving successive aza-Michael addition of an appropriate primary amine in DMSO (which proved superior to DMF, NMP or DCE) and acylation with acryloyl chloride in the presence of triethylamine (Scheme 2). Typically, each elongation cycle reached 2.5 day even for unhindered amines [208].

**Scheme 3:**
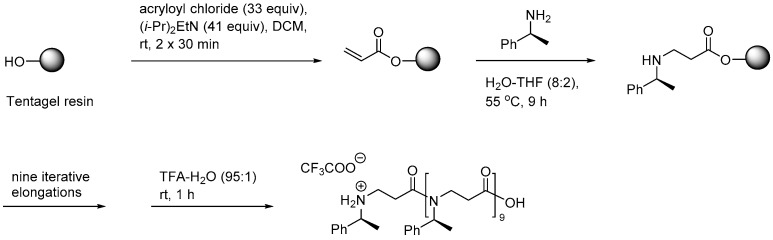
SPS of α-chiral β-peptoids via an optimized submonomer aza-Michael method [209].

Subsequently, SPS of longer β-peptoid oligomers (up to 10 residues; Scheme 3), displaying α-chiral side chains, was achieved by using a Tentagel resin [209]. Although faster coupling cycles of approx. 10 h were applicable when H_2_O–THF (8:2) was employed in the optimized aza-Michael addition, the protocol remained time-consuming as compared to preparation of peptides and α-peptoids.

**Scheme 4:**
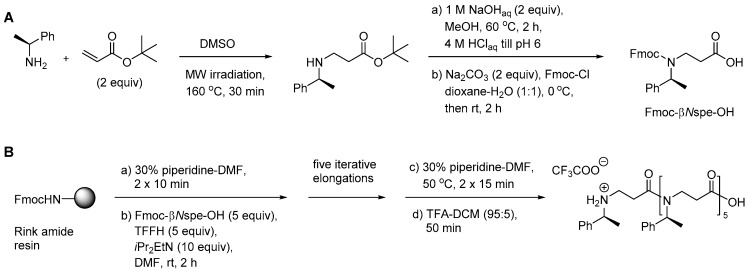
Monomer-based SPS using prefomed α-chiral Fmoc-β*N*spe-OH building blocks [210]; (**A**) synthesis of monomers; (**B**) SPS protocol.

To overcome the incomplete conversion during the aza-Michael addition step in SPS, an alternative method, involving a preformed Fmoc-protected substituted β-alanine monomer, was investigated [210]. In this case the aza-Michael addition to *t*-butyl acrylate was readily accomplished under MW heating to 160 °C in DMSO. Ester hydrolysis, Fmoc protection under standard conditions, followed by column chromatography afforded the required Fmoc-β*N*spe-OH building block (Scheme 4A). Nevertheless, repeated couplings with PyBOP only yielded the full-length oligomer as a minor product, while in situ formation of the acyl fluoride with TFFH proved more efficient (Scheme 4B) [210].

**Scheme 5:**
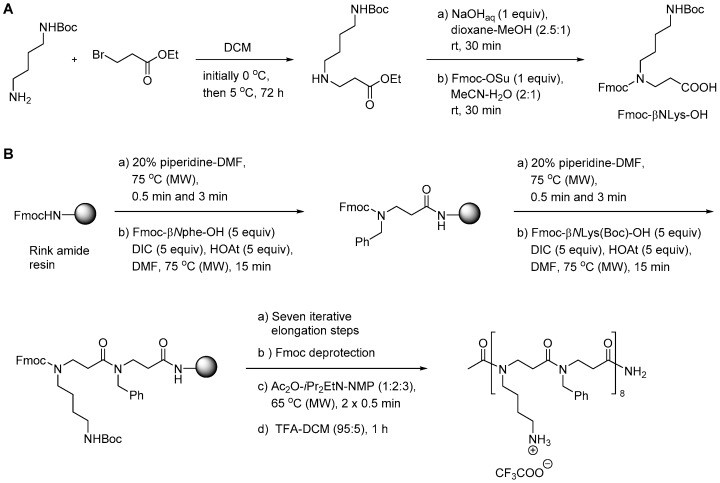
MW-assisted SPS using prefomed Fmoc-protected building blocks [211]; (**A**) synthesis of Fmoc-protected β*N*Lys monomer; (**B**) protocol for assembly of oligomers.

Till now, the most convenient monomer-based protocol for synthesis of longer oligomers involves automated MW-assisted Fmoc-based SPS (Scheme 5) with preformed building blocks (e.g., alternating β-peptoid analogs of Lys and Phe) in combination with DIC/HOAt (standard coupling at 75 °C) [211]. This allowed assembly of a 16-mer in less than 1 day in a yield and purity comparable to that of the corresponding peptide. In this case Fmoc-β*N*Phe-OH was obtained from the aza-Michael adduct by successive treatment with TFA–DCM (1:2) followed by *N*-protection with Fmoc-Cl [211].

**Scheme 6:**
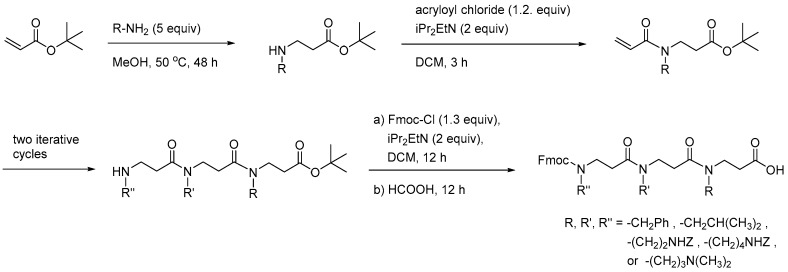
Synthesis of trimeric achiral β-peptoid building blocks [212].

**Scheme 7:**
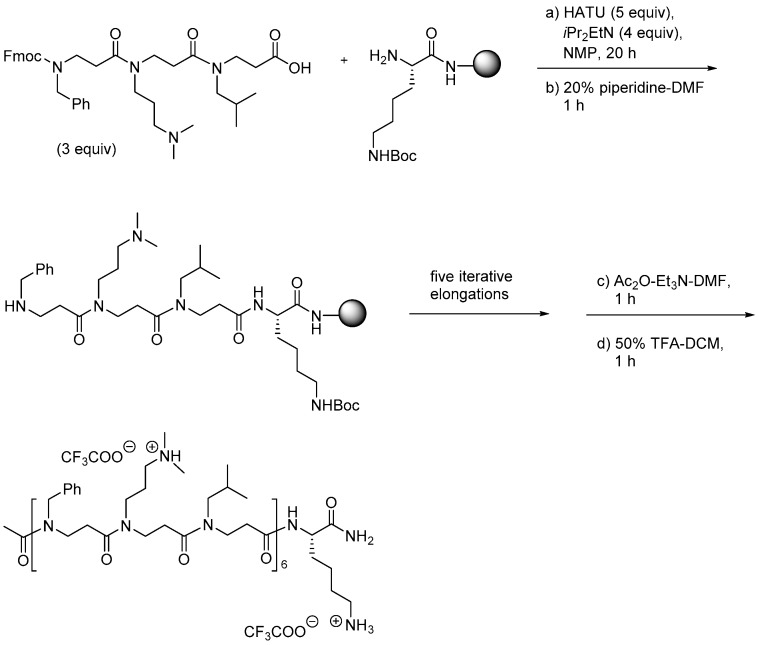
Block SPS of H-[β*N*Phe-β*N*Orn(Me_2_)-β-*N*Leu]_6_-Lys-NH_2_ [212].

Yet another synthesis methodology has been reported by Shuey et al. [212]. Here dimeric and trimeric building blocks were prepared either by a solution-phase route (Scheme 6) or via the SPS method by Hamper et al. [208] by including on-resin Fmoc protection prior to release from resin. A library of peptidomimetics (9–18 residues) was obtained by assembly on a resin preloaded with Fmoc-Lys(Boc)-OH by using HATU/DIEA for amide ligations (Scheme 7).

**Scheme 8:**
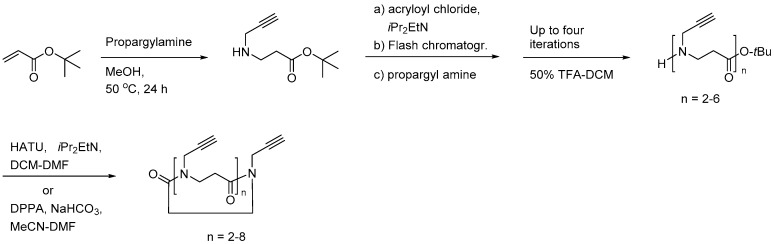
Solution-phase synthesis of cyclic β-peptoids [213].

Also, cyclic β-peptoids, displaying alkyne side chains, allowing for modification by click chemistry, have been prepared by repetitive solution-phase elongations (acylation with acryloyl chloride followed by aza-Michael addition at 50 °C in MeOH) of the starting propargylamine aza-Michael adduct obtained with *t*-butyl acrylate (Scheme 8) [213]. Macrocyclisation of the linear intermediates was accomplished with HATU or diphenylphosphoryl azide (DPPA). However, cyclisation of the linear dimer and trimer (n = 2 or 3) gave rise to rings of doubled size due to an intermolecular elongation occurring prior to cyclisation.

#### 2.3.2. Antimicrobial Activity

Of the 21 members in the array of β-peptoids (9–18 residues besides the C-terminal lysine), reported by Shuey et al., six oligomers showed moderate antibacterial activity corresponding to a MIC value of 128 µg/mL against *E. coli* (ATCC 25922) [212], which for the most active 19-mer oligomer (H-[β*N*Phe-β*N*Orn(Me_2_)-β*N*Leu]_6_-Lys-NH_2_) corresponds to 35 µM. In comparison, a 16-mer oligomer with an alternating cationic/hydrophobic sequence (i.e., Ac-[β*N*Lys-β*N*Phe]_8_-NH_2_) exibited MIC values in the range 4–16 µM against wild-type *E. coli* (ATCC 25922) and three β-lactamase-producing (ESBL, AmpC and NDM-1) strains, whereas it was practically inactive toward *K. pneumoniae* (KPC-2 and NDM-1 strains) and MRSA (MIC values of 256 µM) [211]. Moreover this oligomer resisted enzymatic degradation with pronase and displayed weakly hemolytic properties (HC_10_ > 128 µM) and moderate cytotoxicity against HeLa cells (IC_50_ of 169 µM) [211].

### 2.4. α-Peptide/β-Peptoid Hybrids

As discussed above (Section 2.3.1) SPS of α-chiral β-peptoids proved surprisingly challenging due to sluggish amide formation involving resin-bound congested secondary amines [210], albeit it was later found that achiral oligomers were readily obtained by MW-assisted SPS [211]. In contrast, hybrids with a design of alternating α-amino acid and β-peptoid residues were readily obtained via SPS by using dimeric building blocks synthesized by standard solution-phase methods [214,215]. In subsequent studies these α-peptide/β-peptoid hybrids were found to possess a number of favorable properties: (i) high potency toward MDR bacteria; (ii) proteolytic stability; (iii) low hemolysis and cytotoxicity, and (iv) increased activity in the presence of plasma as opposed to most antimicrobial peptides (AMPs) [113,120,216,217,218,219,220]. In addition, anti-biofilm and antiplasmodial activities have been reported [221,222]. Recently, lipidated analogs were found to exert anti-inflammatory and immunomodulating properties, e.g., neutralization of LPS- and LTA-induced proinflammatory responses by immune cells [223] as well as agonism and antagonism of neutrophilic formyl peptide receptors depending on the nature of the lipid headgroup [224,225].

#### 2.4.1. Synthesis

**Scheme 9:**
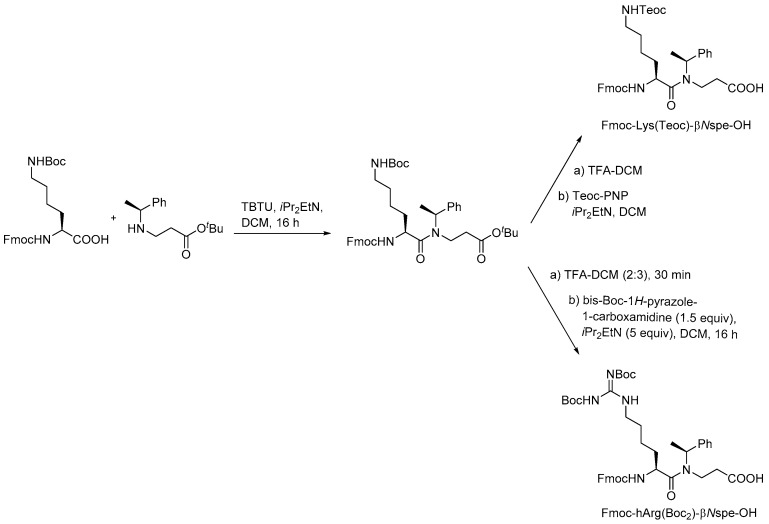
Synthesis of Teoc-protected and guanidinylated dimeric building blocks [215].

In the initial synthetic work on α-peptide/β-peptoid hybrid oligomers with alternating sequences both submonomer and monomer approaches were considered to be inapplicable due to the incomplete conversion obtained in on-resin aza-Michael additions of sterically hindered α-chiral amines as well as the sluggish amide formation involving the resulting even more congested β-peptoid amines as discussed above for SPS of homomeric β-peptoid oligomers involving preformed Fmoc-protected monomers [210]. Instead, versatile gram-scale protocols for obtaining dimeric building blocks were developed. Similar to the synthesis of β-peptoid monomers an aza-Michael addition of the appropriate primary amine to *t*-butyl acrylate produced an *N*-alkylated β-alanine ester that was subjected to coupling with a commercial Fmoc-protected lysine building block to give a dimeric intermediate (Scheme 9). Manipulation of the protection scheme gave access to building blocks displaying an *N*^ε^-(2-trimethylsilylethyl)oxycarbonyl (*N*-Teoc), or a bis(Boc)-guanidino side chain functionality [215].

**Scheme 10:**
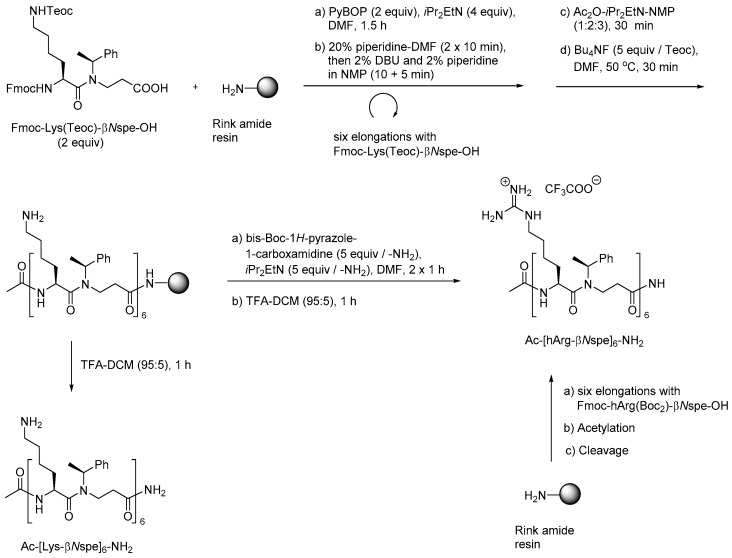
Approaches for assembly of cationic/hydrophobic α-peptide/β-peptoid oligomers [215].

The utility of these building blocks was explored via two routes (Scheme 10) of which the first comprised assembly of an *N*^ε^-Teoc-protected resin-bound oligomer that subsequently was side-chain deprotected with TBAF allowing for on-resin re-functionalization, e.g., to give homoarginine-containing oligomers, which also could be obtained directly by employing the corresponding preformed dimeric building block, i.e., Fmoc-hArg(Boc_2_)-β*N*spe-OH [215].

**Figure 10:**
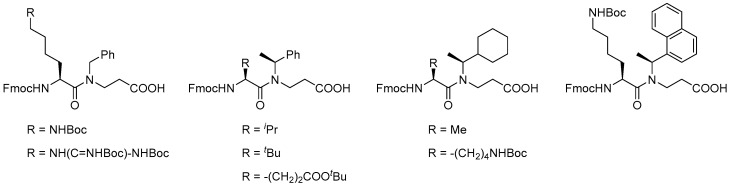
Dimeric building blocks obtained by solution-phase methods [214].

To enable structure-activity studies of this novel class of peptidomimetics, an array of diverse dimeric building blocks comprising variations in hydrophobicity and charge was constructed (Figure 10) [214]. While the MW-assisted (DMSO, 150 °C) aza-Michael addition proved insensitive to sterical hindrance of the amine, the subsequent amide formation with an Fmoc-protected α-amino acid required adjustment of the standard conditions (TBTU, room temperature, 16 h) when one of the reactants was congested. Thus, for coupling of the Fmoc-*^t^*BuGly-OH/H-β*N*spe-OH and Fmoc-Lys(Boc)-OH/H-β*N*sce-OH pairs use of TFFH in 1,2-dichlorothane (DCE) at elevated temperature (MW 60 °C, 0.5 h) was found to give acceptable yields. To accomplish the coupling between the α-chiral naphtylamine intermediate and Fmoc-Lys(Boc)-OH even harsher conditions were needed (TFFH, MW 80 °C, 2 h).

**Scheme 11:**
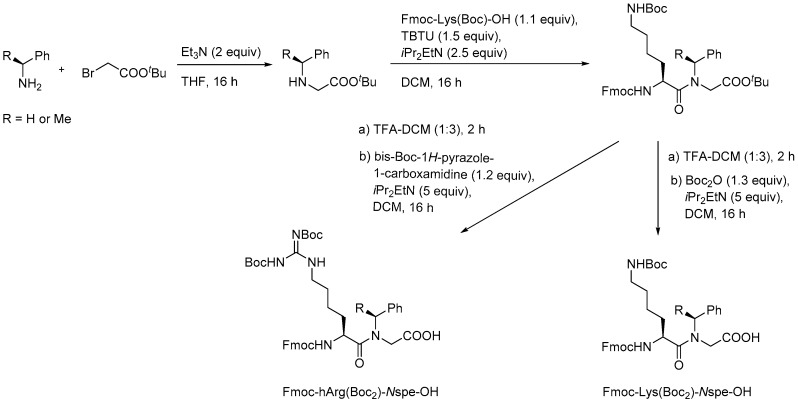
Synthesis of α-peptide/α-peptoid dimeric building blocks [120].

Dimeric α-peptide/α-peptoid building blocks Fmoc-Lys(Boc)-*N*Phe-OH and Fmoc-hArg(Boc_2_)-*N*Phe-OH were similarly prepared for use in manual SPS of oligomers (Scheme 11) [120].

**Figure 11:**
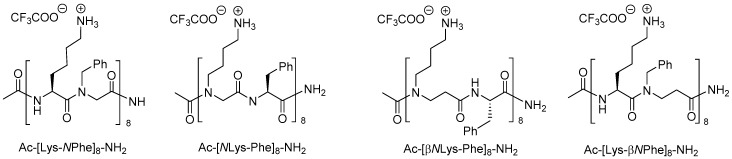
Oligomers assembled by MW-assisted monomer-based SPS [211].

Monomer-based SPS of oligomers consisting of alternating α-amino acids and achiral α- or β-peptoid residues (Figure 11) was feasible when preformed Fmoc-protected monomers were coupled under standard MW-heating conditions (75 °C) used for SPPS [211].

#### 2.4.2. Antimicrobial Activity of α-Peptide/Peptoid Hybrids

In a preliminary brief study by Olsen et al. α-peptide/β-peptoid hybrids (12-mers) with an alternating cationic/hydrophobic design, displaying Lys or hArg in combination with α-chiral β*N*spe or achiral β*N*Phe residues, were tested for antibacterial activity [215]. The oligomer Ac-[hArg-β*N*Phe]_6_-NH_2_ was most potent against both *E. coli* (ATCC 11229) and *S. aureus* (ATCC 6633) with MIC values of 2.9 µM and 11.3 µM, respectively [215].

Next, a clear correlation between increasing oligomer length and antibacterial activity was established for Lys-based hybrids, i.e., Ac-[Lys-β*N*spe]_n_-NH_2_ (n = 5–8), against both Gram-positive (MRSA and VRE) and Gram-negative bacteria (*E. coli* and *P. aeruginosa*) [216]. The 16-mer in this series only displayed high activity against *P. aeruginosa* (MIC of 4.7 µM), thereby appearing to be a potential lead compound with a narrow antibacterial spectrum [216]. A requirement for at least 12 residues to confer activity was seen in two series of hArg-based oligomers, incorporating α-chiral and achiral β-peptoid residues (i.e., Ac-[hArg-β*N*spe]_n_-NH_2_ and Ac-[hArg-β-*N*Phe]_n_-NH_2_). When tested against MRSA (ATCC 33591) Ac-[hArg-β*N*spe]_7_-NH_2_ proved to be the most potent analog (MIC = 2.7 µM), whereas most 12- to 16-mers displayed almost equal MICs (2.5–5 µM) against *E. coli* (ATCC 25922). The general trend is that guanidino-containing compounds are more potent than the corresponding amino-functionalized oligomers (except against *P. aeruginosa*). Introduction of α-chirality in the β-peptoid residues induces a 2- to 4-fold increase in activity toward MRSA, which in part may be ascribed to the concomitant incorporation of several methyl groups, thereby increasing hydrophobicity [216]. Noticeably, even for the 16-mers in each series the hemolytic activity remained low (HC_10_ > 500 µg/mL). In addition, two 12-mers with increased hydrophobicity, obtained via incorporation of α-chiral cyclohexyl-containing β-peptoid units (i.e., β*N*sce), were examined: for Ac-[hArg-β*N*sce]_6_-NH_2_ high antimicrobial activity (e.g., MIC of 1.6 µM against VRE) was accompanied by considerable hemolytic activity (HC_10_ = 25 µg/mL), whereas the mixed analog Ac-[hArg-β*N*sce-Lys-β*N*spe]_3_-NH_2_ had an activity profile similar to that of hArg-based oligomers while retaining low hemolytic activity.

Despite an observed low hemolytic activity independently of length and type of cationic α-amino acid, prehemolytic alterations in the morphological appearance of erythrocytes were more pronounced for longer analogs containing hArg and/or α-chiral β-peptoid residues [216,221]. Comparison of 16-mer oligomers indicated that Lys-based hybrids are 4-fold less cytotoxic than the corresponding hArg analogs when tested against HeLa cells [216].

In another study [217], also including some food-borne bacteria and clinical ESBL-producing *E. coli* strains, Ac-[hArg-β*N*spe]_6_-NH_2_ and Ac-[hArg-β*N*Phe]_6_-NH_2_ exhibited MIC values of 1.5–6 µM against *E. coli* (ATCC 25922 and ESBL-producers), *Listeria monocytogenes*, *Vibrio* spp., *K. pneumoniae* (ATCC 13883) and *S. aureus* (8325). By contrast, Ac-[Lys-β*N*spe]_8_-NH_2_ was less active against *S. aureus* and *K. pneumoniae* (MIC of 19 and 38 µM, respectively). Again, a strong correlation between length and increased potency was observed for a Lys/hArg mixed series: Ac-[Lys-β*N*spe-hArg-β*N*spe]_n_-NH_2_ (n = 2–4) with the 8-mer being weekly active, while the 12-mer was potent (MIC of 3–6 µM) toward most bacteria except *S. aureus* and *K. pneumoniae* (MIC 24 µM), whereas the 16-mer had MIC values of 1–9 µM against most bacteria in the test panel [217]. These findings confirm that approx. 12 residues constitute the minimum length required for substantial antibacterial activity of peptidomimetics belonging to this class. The only bacterium in the test panel that appears to be inherently resistant to these peptidomimetics is *Serratia marcescens* (also resistant to colistin) [217].

Moreover, an ATP leakage assay on *S. aureus* showed that the intracellular ATP content decreased rapidly to a constant low level in the presence of shorter analogs (8- and 12-mers), but was depleted immediately upon exposure to a 16-mer, which infers that the mode of action of these peptidomimetics involves different degrees of membrane permeabilization or disruption depending on the oligomer length [217]. In a model study of membrane interactions a high abundance of guanidino groups (e.g., in Ac-[hArg-β*N*spe]_6_-NH_2_) was found to promote enhanced perturbation (as compared to that of Ac-[Lys-β*N*spe]_6_-NH_2_) of monolayers of a typical outer-leaflet constituent (1,2-dipalmitoyl-*sn*-glycero-3-phosphatidylglycerol; DPPG) of Gram-positive cell membranes. Interestingly, there was no significant difference in the ability of the hArg- and Lys-based oligomer to interact with lipopolysaccharide Kdo2-lipid A monolayers mimicking the surface layer of Gram-negative cell envelopes [226]. A proposed interpretation is that both amino- and guanidino-containing compounds may have similar self-promoted mechanism of penetrating the LPS layer via displacement of bivalent metal cations with an ensuing destabilization of the integrity of the LPS layer. As a result of this the relative antibacterial activity of these two subtypes of peptidomimetics against Gram-negative bacteria appears to rely on their different ability to compromise the integrity of the inner membrane [226]. A direct comparison of Ac-[Lys-β*N*spe]_6_-NH_2_ and Ac-[hArg-β*N*spe]_6_-NH_2_ against a panel of pathogens showed that the hArg-based oligomer was 2- to 8-fold more active against Gram-negative species while it had 4- to 16-fold higher potency toward Gram-positive bacteria [226], which further corroborates previous findings that guanidino groups promote activity against Gram-positive bacteria [215,216,217].

*Staphylococcus epidermidis* is a common cause of nosocomial infections associated with surface colonization of indwelling devices such as catheters. Hence, an array of peptidomimetics, representing different lengths of subsets of oligomers displaying either Lys, hArg or Lys/hArg as the cationic residues, was tested against planktonic and biofilm cultures of methicillin-resistant *S. epidermidis* [222]. For Lys-containing subclasses Ac-[Lys-β*N*spe]_n_-NH_2_ (n = 10–16) and Ac-[Lys-β*N*spe-hArg-β*N*spe]_n_-NH_2_ (n = 1–4) the MIC values against planktonic bacteria decreased markedly with increasing oligomer length (MICs in the range 0.3–2.1 µM for 12-mers and longer analogs). On the other hand, all members of hArg-based subclasses, i.e., Ac-[hArg-β*N*spe]_n_-NH_2_ and Ac-[hArg-β*N*Phe]_n_-NH_2_ (n = 5–8), displayed high activity (MICs in the range 0.4–1.8 µM) with the dodecamers being the most potent. Time-kill experiments (at 2 or 4 × MIC) revealed that fast-growing *S. epidermidis* was efficiently killed within 1–5 h by all tested subtypes, while bactericidal effect on slow-growing stationary-phase cells required 3–24 h depending on oligomer length and subtype. Importantly, at their MICs (measured in biofilm medium) representatives of all the above subtypes prevented formation of *S. epidermidis* biofilm, with Ac-[hArg-β*N*spe]_6_-NH_2_ being the most efficient, since treatment with 1.4 µM led to 100% reduction of biomass. However, eradication of young (6-h-old) biofilm could only be achieved at concentrations 10 to 20 times the MIC, whilst complete killing of mature (24-h-old) biofilm required even higher concentrations [222]. In order to assess their potential clinical utility, the effect of 12- and 16-mers on cell viability was determined in HeLa cells, and the two dodecamers Ac-[Lys-β*N*spe]_6_-NH_2_ and Ac-[Lys-β*N*spe-hArg-β*N*spe]_3_-NH_2_ were found to be least toxic (IC_50_ >1000 µM and 295 µM, respectively). Lack of α-chirality in Ac-[hArg-β*N*Phe]_6_-NH_2_ was beneficial, since it was 3-fold less cytotoxic as compared to Ac-[hArg-β*N*spe]_6_-NH_2_ (IC_50_ values of 96 µM versus 32 µM) [222].

A follow-up study elaborated on the factors determining antibacterial activity versus cytotoxicity for α-peptide/α-peptoid and α-peptide/β-peptoid oligomers displaying Lys and hArg residues as alternating cationic components [120]. An array of such achiral and α-chiral hybrids with oligomer lengths of 10, 12 and 14 residues was examined. All designs conferred high activity (MICs in the range 1–4 µM) toward *E. coli* (ATCC 25922 and MDR strains) for oligomers with a minimum length of 12 residues. For *A. baumannii* a more clear length-activity correlation was observed with ~4-fold increase in potency by each elongation with a dimeric unit (with the 14-mer Ac-[Lys-*N*spe-hArg-*Ns*pe]_3_-Lys-*N*spe-NH_2_ being the most active: MIC = 1 µM); a similar trend was seen for VRE (with Ac-[Lys-*N*spe-hArg-*N*spe]_3_-NH_2_ displaying highest potency: MIC = 2 µM). By contrast, a generally reduced potency (MICs in the range 8–128 µM) was observed against *S. aureus* [120]. Investigation of cytotoxic side effects of AMPs has often been confined to evaluation of hemolytic properties, however, this study infers that other relevant cell lines need to be considered as well, since even essentially non-hemolytic compounds were found to exert detrimental effects on e.g., HepG2, NIH 3T3 and human umbilical vein endothelial cells (HUVEC) at remarkably low concentrations [120,211]. In particular, HeLa cells were found to be extremely susceptible to longer oligomers: a 2.5- to 3.5-fold lowering of cell viability was seen for each added dimeric unit resulting in IC_50_ values below 100 µM for all 14-mers, whereas 1.5- to 2-fold increments in cytotoxicity were seen for other cell lines with endothelial HUVEC being the most resistant benign cell type (IC_50_ above 350 µM for all 12-mers). Cytotoxicity of α-peptide/β-peptoid hybrids was only slightly increased as compared to that of the corresponding α-peptoid-containing hybrids despite the presence of additional methylene units in the backbone, thus inferring that the number of side chains is more important than the distance between the *N*- and *C*-termini. Moreover, introduction of α-chiral side chains with concomitantly increased hydrophobicity resulted in more than 3-fold lowered viability (in terms of IC_50_ values) for all tested cell lines [120]. When targeting *E. coli* strains the best cell selectivity index (SI = the mean IC_50_ divided by MIC) was found for the 12-mers Ac-[Lys-*N*spe-hArg-*N*spe]_3_-NH_2_ and Ac-[Lys-β*N*spe-hArg-β*N*spe]_3_-NH_2_ (SI > 450), while for *A. baumannii* the 14-mer analogs displayed best cell selectivity (SI > 65). Thus, by careful optimization of oligomer length, presence/absence of peptoid chirality (and thus varying hydrophobicity and/or folding propensity), and balancing the ratio between Lys and hArg, potential lead compounds against MDR *E. coli* strains were identified [120].

Yet another optimization strategy, involving modification of the N-terminus of these peptidomimetics, has been explored [218]. Introduction of *N*-terminal hydrophobic acyl moieties in Ac-[Lys-β*N*spe-hArg-β*N*spe]_3_-NH_2_, displaying only a moderate activity against MDR Gram-positive bacteria (MIC = 16 µM against MRSA and VRE [120]) led to remarkably increased activity against MRSA and VRE with typical MIC values of 1–2 µM. Interestingly, the potency against *E. coli* (ATCC 25922 and MDR strains) was unaffected by modification with polycationic, PEG-like, or hydrophobic (aliphatic, aromatic and fluorous) moieties, whereas the activity toward *A. baumannii* was improved 4- to 8-fold (to MICs of 1–2 µM) by appending polycationic or hydrophobic/fluorous moieties [218]. Increasing the cationicity via modification with spermine-acetyl was beneficial for the activity toward MRSA and VRE (4- and 16-fold improved MICs). Despite increased cytotoxicity against NIH 3T3 cells and HUVEC, the optimized peptide analogs exhibited promising cell selectivity (SI ~400–600). However, it is striking that even though several analogs displayed similar cytotoxicity against HUVEC and NIH 3T3 cells, they exhibited quite different hemolytic properties. For example, adamantylacetyl and decanoyl derivatives had 4- to 10-fold lower HC_50_ as compared to the compounds modified with the overall most favorable hydrophobic activity-inducing (against Gram-positive bacteria) cyclohexylacetyl and pentafluorophenylacetyl groups. Interestingly, the hemolytic properties could be correlated to the hydrophobicity as measured by retention on reversed-phase analytical HPLC (C_18_ column), where a certain acetonitrile concentration at peak elution could be defined as the upper threshold below which hemolysis was negligible. Hence, end group modification constitutes a powerful tool for improving potency of a moderately active compound, albeit it is necessary to include a thorough investigation of possible cytotoxic side effects [120,218].

The impact of the backbone design of peptidomimetics was explored by testing an array comprising 16-mer α-peptides, β^3^-peptides, α- and β-peptoids as well as hybrids with alternating α-amino acids and unnatural residues. All compounds shared a common sequential display of cationic and hydrophobic side chains (Lys- and Phe-like) [211]. Test against four *E. coli* (ATCC 25922 and MDR) strains revealed that hybrids, consisting of alternating sequences of α-amino acids and unnatural residues, exhibited superior antibacterial activity as compared to the α-peptides (very hemolytic and cytotoxic), β^3^-peptides and both types of peptoids. The two most active hybrids were Ac-[Lys-*N*Phe]_8_-NH_2_ and Ac-[Lys-β*N*Phe]_8_-NH_2_ (MICs of 2–8 µM) that both were weakly hemolytic (HC_10_ > 128 µM), albeit the α-peptoid-containing analog was less cytotoxic against HeLa cells than the slightly more hydrophobic β-peptoid-based analog (IC_50_ of 100 and 26 µM, respectively) [211]. Such comparative investigations of different types of backbones may in fact constitute a powerful general optimization strategy although most previous work on antibacterial peptidomimetics concern specific subclasses.

A study focused on Ac-[Lys-*N*Phe]_8_-NH_2_ showed that it exhibited activity against a range of Gram-negative pathogens (MICs in the range 0.2–10 µM) [109]. Time-kill experiments at its MIC showed that killing was surprisingly slow (>3 h) including a lag period of approx. 1 h. This infers that alternative mechanisms involving interference with intracellular processes are likely to contribute significantly to the killing at the MIC, while a mode of action involving outer membrane permeabilization and partial depolarization of the inner membrane appeared to be dominating at higher concentrations. Also, prevention of biofilm formation in several Gram-negative bacteria was seen even at subMIC levels. Moreover, after continuous exposure of *E. coli* to subinhibitory concentrations this peptidomimetic proved less prone to induce resistance development as compared to standard antibiotics (e.g., ciprofloxacin, gentamicin, cefotaxime and imipenem). Another intriguing feature of Ac-[Lys-*N*Phe]_8_-NH_2_ is its anti-inflammatory effect, which proved similar to that of IDR-1018 [227] with respect to inhibition of the LPS-induced pro-inflammatory response of neutrophils (IL-6 and TNF-α secretion was abolished in the presence of 20 µg/mL of both compounds) [109]. Overall, the antibacterial effect of this peptidomimetic appears to involve multiple mechanisms with a simultaneous prevention of exacerbated immune response to pro-inflammatory components released from Gram-negative pathogens upon killing [109].

Most recently, 21 variants of H-[Lys-β*N*Phe]_16_-NH_2_ were investigated with emphasis on the influence of hydrophobicity, fluorination and distribution of cationic/hydrophobic residues on antibacterial activity against Gram-positive and Gram-negative bacteria versus hemolytic properties and cytotoxicity [219]. The array comprised subsets of analogs (12- and 16-mers) displaying: (i) variable side chain length of cationic residues, (ii) substitution in the aromatic β-peptoid units, and (iii) alternative distribution patterns of cationic/hydrophobic residues. An interesting correlation between oligomer length and activity toward the Gram-positive *S. pseudintermedius* (a major pathogen associated with infections in dogs) was observed, since most 12-mers were either equally or more potent (up to 4-fold lower MICs) than the corresponding 16-mers. Shortening of the cationic side chain (e.g., Lys → Dab) conferred slightly increased hydrophobicity that led to an unexpected decrease in activity against *S. pseudintermedius*, whereas the presumed opposite tendency was found for the other Gram-positive bacteria tested (i.e., *S. aureus* and VRE); this modification also induced hemolytic properties (HC_10_ = 19 µM) [219]. Introduction of hydrophobicity-increasing substituents (Me, one or three F atoms or a CF_3_ group) likewise conferred increased potency against Gram-positive bacteria, but the concomitant enhancement of hemolytic properties was considerably more pronounced except for the analogs displaying a single fluorine at the 4-position in the β-peptoid residues. In particular this 16-mer displayed promising activity towards both Gram-positive and Gram-negative species (MICs of 0.8–1.6 µM and 1.6–3.2 µM, respectively). However, it affected the viability of NIH 3T3 cells (IC_50_ = 132 µM) more than the 4-Me substituted analog (IC_50_ = 527 µM), albeit the latter was much more hemolytic (HC_10_ = 2.6 µM), thus again highlighting the discrepancy between hemolysis and cytotoxicity toward other mammalian cells. In addition, comparison of the different arrangements of cationic and hydrophobic residues (alternating, pairwise and complete segregation at each terminus) revealed that analogs with an alternating display possess the overall most favorable activity profiles [219]. These findings confirm that for this class of peptidomimetics hydrophobicity must be kept below a certain threshold level to retain satisfactory cell selectivity. Indeed a low degree of fluorination appears to be beneficial as it promotes general antibacterial activity with only a minor effect on toxicity toward mammalian cells [219]. Table 6 shows the activity profiles for some of the most active α-peptide/β-peptoid hybrids.

Intriguingly, the antibacterial activity of α-peptide/β-peptoid peptidomimetics (e.g., Ac-[hArg-β*N*Phe]_6_-NH_2_) against *E. coli* was found to be potentiated by the presence of plasma with a typical lowering of the MICs by one order of magnitude, whereas testing in medium containing heat-inactivated plasma led to slightly lowered potency. A similar effect was observed for a membrane-active AMP, while the activity of two AMPs with intracellular targets was unaffected by plasma [220]. A similar enhancement effect of plasma (but not of serum) on the activity of these peptidomimetics was subsequently observed for a range of pathogenic Gram-positive and Gram-negative bacteria [113]. Typically, both MICs and MBCs were 2- to 16-fold lower when obtained at a plasma concentration that in itself did not exert membrane disruption as visualized by scanning electron microscopy [113]. Consequently, the traditional MIC determinations in growth medium (e.g., MHB) used for initial evaluation of the clinical relevance of a given membrane-active compound may in fact overestimate the concentrations required for in vivo proof-of-concept experiments. Although *E. coli* mutants with stable resistance could be selected for by adaptive evolution experiments [110], testing of these in the presence of plasma resulted in MICs of the peptidomimetics similar to those found against the original wild-type strain used in the adaption experiment [110]. Moreover, the acquisition of resistance towards these peptidomimetics seem to be specific as it did not confer cross-resistance to a number of AMPs including LL-37, which is part of the human innate immune defense [110]. Thus, in combination with the favorable synergistic interactions with plasma components these findings significantly increase the therapeutic potential of these peptidomimetics.

While Ac-[Lys-*N*Phe]_8_-NH_2_ exerted anti-inflammatory effects at concentrations of 5–10 µM [109], lipidated analogs of Ac-[Lys-β*N*spe]_8_-NH_2_ blocked LPS-induced cytokine secretion from human leukocytes at nanomolar concentrations comparable to the effect of polymyxin B [223]. Additionally, these fatty acid-conjugated peptidomimetics were capable of neutralizing the response to LTA, which constitutes a major pro-inflammatory membrane component of Gram-positive bacteria. Overall the most promising compound was Pam-[Lys-β*N*spe]_6_-NH_2_: for LPS and LTA neutralisation the IC_50_ values measured for IL-6 secretion were 60 nM and 0.85 µM, respectively; with a selectivity index of 400 based on its cytotoxicity toward leukocytes. This immunomodulatory activity is dependent on a high content of Lys residues and acylation with a long-chain fatty acid (e.g., palmitoylation) as well as on sufficient oligomer length (at least 12 residues). In contrast to many lipidated AMPs these peptidomimetics exhibited lower antibacterial activity (MICs of 8–16 µM) as compared to the parent oligomer [223]. Moreover, Pam-[Lys-β*N*spe]_6_-NH_2_ was recently identified as a promising selective inhibitor of human neutrophil activation via formyl peptide receptor 2 (FPR2) [225]. Thus, FPR2-induced NADPH-oxidase responses (e.g., superoxide release and degranulation) were antagonized by Pam-[Lys-β*N*spe]_6_-NH_2_ with a potency (IC_50_ = 50 nM) similar to that of the previously most efficient peptide, the gelsonin-derived PBP10 (IC_50_ = 60 nM) [225]. The related analog Lau-[Lys-β*N*spe]_6_-NH_2_ was found to be a potent antagonist of the mouse orthologue Fpr2, thereby establishing these compounds as the first class displaying selective cross-species activity, which enable exploration of the role of FRP2 in regulation of immunological processes via mouse models [228]. This novel class of selective FPR2 antagonists constitutes potential lead compounds for development of novel anti-inflammatory drugs. A SAR study of an array of lipidated analogs displaying a headgroup with two hydrophobic tails led to identification of Lau-[(S)-Aoc]-[Lys-β*N*Phe]_6_-NH_2_ as a potent FPR2 agonist capable of activating both human and mouse neutrophil NADPH-oxidase (EC_50_ of 167 nM and 82 nM, respectively) [224]. The structural requirements for potent agonistic interaction proved to be very specific: (i) analogs displaying slightly shorter or longer fatty acids, e.g., decanoyl or myristoyl instead of lauroyl, had significantly reduced potency, (ii) replacement of (*S*)-2-aminooctanoic acid (Aoc) with analogs having shorter/longer alkyl side chains were devoid of activity, and (iii) incorporation of (*R*)-Aoc also compromised activity [224]. This subclass of lipidated α-peptide/β-peptoid hybrids appears to constitute a valuable tool for future studies of FPR2 signalling in vivo as well as a source of potential leads for development of prophylactic anti-infectives that stimulate neutrophils towards enhanced phagocytosis of bacteria.

### 2.5. β^3^-Peptides

**Figure 12:**
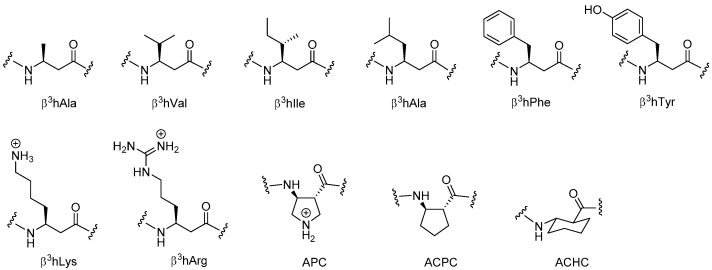
Typical β^3^-amino acid residues in designed antibacterial and antifungal β^3^-peptides.

β^3^-Peptides constitute interesting antimicrobial candidates as they may possess stable secondary structures and are resistant to proteolytic degradation. In fact three distinct helical conformations have been identified for β-peptides, with the helix type depending on the substitution pattern on the β^3^-amino acid residues [229,230,231]. The early work on antibacterial β^3^-peptide oligomers comprise contributions by the groups of DeGrado in 1999 [232], Gellman in 2000 [233], and of Seebach in 2001 [230]. In general β^3^-amino acids were assembled by standard Fmoc-based SPS; in Figure 12 some typical examples of residues are shown, while the synthesis of the building blocks will be briefly discussed below.

#### 2.5.1. Synthesis of β^3^-Amino Acid Building Blocks

**Scheme 12:**
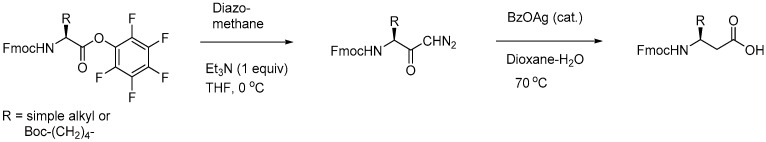
Arndt-Eistert reaction of pentafluorophenyl esters with subsequent Wolff rearrangement of the α-aminoacyldiazomethane intermediate to afford Fmoc-protected β^3^-amino acid building blocks [234].

Although several suitably protected β^3^-amino acid building blocks are commercially available they are usually exceedingly expensive due to their often quite elaborate total synthesis. However, simple acyclic compounds may be obtained via homologation of α-amino acid derivatives, e.g., a convenient synthesis is based on Fmoc-protected pentafluorophenyl esters as depicted in Scheme 12 [234]. This procedure is chromatography-free and allows for the gram-scale preparation of building blocks corresponding to the acyclic residues depicted in Figure 12.

**Scheme 13:**
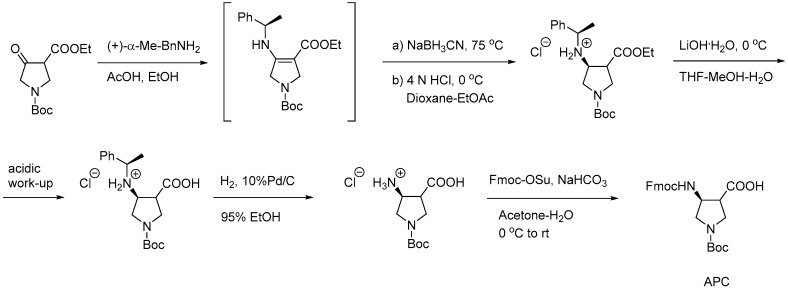
Synthesis of an Fmoc-protected cyclic β^3^-amino acid building block [236].

The building blocks corresponding to the cyclic residues APC and ACPC may readily be obtained in multigram scale from the corresponding commercially available β-keto ethyl esters by similar routes [235,236]. The synthetic sequence starts (shown for APC in Scheme 13) with a conversion into the corresponding enamine upon reaction with (*R*)-α-methylbenzylamine in the presence of acetic acid, followed by reduction in situ with sodium cyanoborohydride. The desired major isomer may be obtained as the corresponding hydrochloride in diastereomerically pure form via a two-step crystallization protocol from the mixture of four diastereomers produced. The subsequent alkaline ester hydrolysis, hydrogenolytic removal of the (*R*)-α-methylbenzyl group, and Fmoc protection of the resulting amine can be performed in rapid succession to give the final product that can be purified by crystallization [236].

#### 2.5.2. Antibacterial Activity of β^3^-Peptides

As only a very few works on the antibacterial activity of β^3^-peptides have been reported during the last decade a brief account of the essential early findings is given together with the most recent contributions. The initial series of antimicrobial β^3^-peptides reported by Hamuro et al. proved to be too hydrophobic to possess any cell selectivity for bacteria over erythrocytes [232]. Subsequently, it was shown that more polar analogs (e.g., H-[β^3^-hAla-β^3^hLys-β^3^-hVal]_n_-NH_2_, n = 4 or 5) displayed lower antimicrobial activity (MICs of 26 and 20 µg/mL, respectively), but with significantly less concomitant hemolytic properties (HC_50_ values of 910 and 320 µM) [237]. In a related series of oligomers (*H*-[β^3^hAla-β^3^hLys-β^3^hPhe]_n_-OH, n = 3–6) only the 12-mer showed moderate activity against *E. coli* and *K. pneumoniae* (MICs of 32–64 µg/mL) [230].

In the seminal work on amphipathic antibacterial β^3^-peptides based on the constrained (*3R*,*4S*)-*trans*-4-aminopyrrolidine-3-carboxylic acid (APC) and (*R*,*R*)-*trans*-2-aminocyclopentane-carboxylic acid (ACPC), a 17-residue 12-helical oligomer (denoted β-17: Ac-[ACPC]_2_-[APC-ACPC-APC-ACPC-ACPC]_3_-NH_2_) was found to possess high antibacterial activity against *E. coli* (MIC = 6.3 µg/mL), *E. faecium* (MIC = 12.5 µg/mL) and *S. aureus* (MIC = 3.2 µg/mL) [238], while it was significantly less hemolytic (HC_50_ ~80 µg/mL) [238].

Although the non-hemolytic (β^3^hArg)_8_-NH_2_ is devoid of antibacterial activity (against a panel of bacteria) it is able to penetrate the envelopes of *E. coli* and *Bacillus megaterium* [239], since confocal laser scanning microscopy (CLSM) of cells exposed to the 7-nitrobenzo-2-oxa-1,3-diazole (NBD)-labeled β^3^-peptide showed a homogeneous intracellular distribution. Moreover, as NBD is a quenchable fluorescent probe it was possible to distinguish the internalized β^3^-peptide from the surface-bound fraction by comparing the fluorescence intensity before and after addition of quenching agent (excess potassium iodide) [239].

Four lipo-β^3^-peptides displaying palmitoyl (Pam) and lauroyl (Lau) moieties (i.e., Pam-β^3^hLys-X-X-β^3^hLys-NH_2_, X = β-Ala, β^3^hAla, β^3^hLys; and Lau-β^3^hLys-β^3^hLeu-β^3^hLeu-β^3^hLys-NH_2_) were compared with the α-peptide analogs, including partially D-amino acid substituted lipopeptides, with respect to their activities toward a panel of Gram-positive and Gram-negative bacteria [240]. Only minor differences were observed between the lipo-β^3^-peptides and lipo-α-peptides as well as analogs containing a single D-α-amino acid [240]. Generally, Gram-positive bacteria proved more susceptible to these lipopeptides than Gram-negative bacteria, of which only *E*. *coli* strains proved moderately susceptible (MICs of 16–32 µg/mL). The most active β^3^-peptide was Pam-[β^3^hLys]_4_-NH_2_ displaying MIC values in the range 4–32 µg/mL against the Gram-positive *Enterococcus* spp., *S. aureus* and *S. epidermidis* (including MRSA and MRSE), with the latter being most susceptible, however, no toxicity data were reported [240].

#### 2.5.3. Antifungal Activity of Helical β^3^-Peptides

In the last decade most research on antimicrobial β^3^-peptides concern the design of short oligomers (9- and 10-mers) displaying *trans*-2-aminocyclohexanecarboxylic acid (ACHC) residues. Analysis by CD has shown that these oligomers typically adopt a 14-helical secondary structure (i.e., 14 atoms in the hydrogen-bonded ring). In the search for novel potential antifungal agents, these relatively short β^3^-peptides (9–10 residues) have been extensively explored with particular focus on activity against *Candida albicans* and other pathogenic *Candida* species. *Candida albicans* is a commensal that may become an opportunistic pathogen causing life-threatening infections (e.g., mucosal candidiasis and invasive candidemia) especially in immunocompromised patients.

First, it was shown that designed 14-helical β^3^-peptides exhibit considerable antifungal properties under CLSI-recommended conditions (i.e., physiological pH and ionic strength) where host-defense α-peptides are inactive against fungal pathogens [241]. From an array of seven β^3^-peptides, the decamer H-β^3^hTyr-[ACHC-β^3^hVal-β^3^hLys]_3_-NH_2_ was identified as the most favorable compound displaying a reasonably low MIC (16 µg/mL) combined with moderate hemolytic properties (21% at the MIC) [241]. The 14-helix has ca. three residues per turn, and hence the repeating triad motif is expected to induce a globally amphipathic structure, in which the hydrophobic ACHC and β^3^hVal residues are clustered on one side of the helix, while the cationic β^3^hLys residues are displayed on the opposite face. This class of antifungal agents may by virtue of its modular structure readily be designed towards improved activity. In a follow-up study, the antifungal activity of 14-helical β^3^-peptides was examined against both planktonic cells and biofilms [242]. Here, it was found that a non-amphipathic analog with a scrambled sequence was devoid of activity, while removal of the *N*-terminal β^3^hTyr residue afforded a significantly less hemolytic compound (HC_10_ ~100 µg/mL) with only slightly reduced antifungal activity (MIC 16–32 µg/mL). This truncated sequence was modified with a 7-methoxycoumarin fluorophore label via a short PEG linker without loss of activity, thereby enabling the use of confocal microscopy to study the interaction with *C. albicans*. It was found that the labelled β^3^-peptide was distributed throughout the cytoplasm of primarily metabolically inactive cells inferring that its mode of action involves membrane disruption. Generally, significantly increased concentrations were required for killing of established biofilms (e.g., 512 µg/mL for coumarin-linker-[ACHC-β^3^hVal-β^3^hLys]_3_-NH_2_), however, at a concentration near its MIC this β^3^-peptide completely prevented planktonic *C. albicans* cells from forming a biofilm, suggesting that β^3^-peptides may be promising agents for preventing fungal colonization [242].

In order to further explore SARs of amphipathic 14-helical β^3^-peptides an array of 25 compounds was designed to represent variations in hydrophobicity and helicity. This was ensured by including several simple hydrophobic β^3^-amino acids (e.g., β^3^hAla, β^3^hVal, β^3^hIle, and β^3^hPhe) as well as the helix-stabilizing cyclic ACHC [243]. Hydrophobicity and helicity of the β^3^-peptides were estimated by their retention in RP-HPLC and by their CD spectra, respectively. The presence of an ACHC residue in each triad of a putative 14-helix was found to be a prerequisite for a high degree of helicity, and at constant helicity increased hydrophobicity was associated with both increased antifungal and hemolytic activity. However, it was possible to identify a window of hydrophobicity in which the corresponding compounds exhibited both high antifungal activity and cell selectivity. Importantly, a detailed analysis of the data revealed a cooperative relationship between helicity and hydrophobicity that determines the antifungal and hemolytic activities of β^3^-peptides [243]. The most active and selective β^3^-peptides identified were H-[ACHC-β^3^hVal-β^3^hLys]_3_-NH_2_ and the corresponding analog displaying β^3^hArg instead of β^3^hLys residues, which both exhibited an MIC of 8 μg/mL and less than 3% hemolysis at their MICs [243].

Next, an array of sixteen 14-helical β^3^-peptides were found to be active against drug-resistant strains and clinical isolates *of C. albicans* as well as three other opportunistic *Candida* species, namely *C. glabrata*, *C. parapsilopsis* and *C. tropicalis* [244]. The three most active compounds (H-[ACHC-β^3^hVal-β^3^hLys]_3_-NH_2_, H-[ACHC-β^3^hVal-β^3^hArg]_3_-NH_2_ and H-[ACHC-β^3^hPhe-β^3^hLys]_3_-NH_2_) displayed MICs of 8–16 µg/mL against the MDR *C. albicans* strains and MICs of 2–32 µg/mL against the panel of *Candida* spp. with the most hydrophobic compound (i.e., H-[ACHC-*β*^3^hPhe-*β*^3^hLys]_3_-NH_2_) being the most active (MICs of 2–8 µg/mL). *C. tropicalis* and *C. glabrata* proved to be the most and least susceptible species, respectively [244]. Generally, an inverse relationship between hydrophobicity of a β^3^-peptide and its MIC was seen for all *Candida* species, and a similar trend was seen for the hemolytic properties of the increasingly hydrophobic analogs H-[ACHC-β^3^hVal-β^3^hLys]_3_-NH_2_, H-[ACHC-β^3^hVal-β^3^hArg]_3_-NH_2_ and H-[ACHC-β^3^hPhe-β^3^hLys]_3_-NH_2_, displaying HC_50_ values of 161, 77 and 15 µg/mL, respectively. The MIC of H-[ACHC-β^3^hVal-β^3^hLys]_3_-NH_2_ against 48-h-old *C. albicans* biofilms was 128 μg/mL, whereas prevention of biofilm formation could be achieved at the MIC against planktonic cells (i.e., at 16 µg/mL); similar results were obtained for the most active β^3^-peptides against *C. glabrata*, *C. parapsilopsis* and *C. tropicalis* biofilms [244].

Due to the relatively high toxicity of common antifungal drugs and the emerging resistance towards these, combination therapy appears as an appealing approach, which was explored by combining H-[ACHC-β^3^hVal-β^3^hLys]_3_-NH_2_ with fluconazole or ketoconazole [245]. Synergistically enhanced antifungal activity of these azoles was obtained with subMIC concentrations of β^3^-peptide and azole, which proved efficient even against fluconazole-resistant *C. albicans* planktonic cells and azole-resistant biofilms; this dual treatment proved to be fungicidal after treatment for 48–72 h. In vitro cytotoxicity of the β-peptide/azole combination was evaluated toward human HepG2 cells, for which viability was not significantly affected at the synergistic concentrations [245].

Recently, cytotoxicity of various antifungal β-peptides was investigated in vitro in the Caco-2 and HepG2 mammalian cell lines [246]. In contrast to observations made for AMPs and antibacterial peptidomimetics, the most hydrophobic and relatively more hemolytic member tested (i.e., β^3^hTyr-[ACHC-β^3^hPhe-β^3^hLys]_3_-NH_2_) was the least cytotoxic toward Caco-2 cells, while the most hydrophilic β^3^-peptide (i.e., H-[ACHC-β^3^hVal-β^3^hLys]_3_-NH_2_) was least tolerated (IC_50_ values of 53.2 and 27.3 µM, respectively). For HepG2 no clear trend was observed except that H-[ACHC-β^3^hVal-β^3^hLys]_3_-NH_2_ despite its higher polarity affected viability most (IC_50_ of 38.8 µM) [246]. Hence, the cell selectivity (ratio between IC_50_ and MIC) was in the range of 2–8 for the four tested compounds also comprising H-[ACHC-β^3^hVal-β^3^hArg]_3_-NH_2_ and β^3^hTyr-[β^3^hVal-β^3^hVal-β^3^hArg]_3_-NH_2_, which is considerably lower (~10- to 100-fold) than usually seen for optimized AMPs and antibacterial peptidomimetics.

Catheter-associated urinary tract infection (CAUTI) constitutes a common type of hospital-acquired infection that may be caused by *C. albicans* biofilm formation on the surfaces of urinary catheters. In view of the above-mentioned ability of antifungal β^3^-peptides to kill *C. albicans* and prevent its biofilm formation under CLSI conditions [242,244], it was of interest to elucidate whether these properties were retained in the presence of urine, being a prerequisite for their potential clinical use in preventing fungal CAUTI. In a model study, a fluorescent β^3^-peptide (i.e., coumarin-linker-[ACHC-β^3^hVal-β^3^hLys]_3_-NH_2_ [242]) was shown to remain as a stable 14-helix (inferred by CD) and to exhibit antifungal and anti-biofilm activity against *C. albicans* in a synthetic medium mimicking the composition of urine [247]. The β^3^-peptide exhibited substantial antifungal activity in synthetic urine with a MIC of 16 µg/mL that was only 2-fold higher than its MIC under standard planktonic susceptibility testing conditions in RPMI medium. These promising findings prompted an investigation of whether this β^3^-peptide could be loaded into polymeric thin film coatings on the intraluminal walls of urinary catheters, thereby creating a surface resistant to *C. albicans* colonization. In fact it proved possible to obtain such polymer-modified polyethylene, polyurethane, and silicone catheters that were capable of continuously releasing β^3^-peptide into the intraluminal space [247]. Catheters coated with β^3^-peptide-loaded polymer multilayers could eradicate planktonic fungal cells for up to 21 days of intermittent challenges with *C. albicans*, and prevent biofilm formation for at least 48 h. This approach holds promise for development of new catheter materials that may reduce the incidence of fungal CAUTIs. In fact, coatings constructed from hyaluronic acid (HA) and chitosan (CH) as well as coatings made-up of poly-l-lysine (PLL) and poly-l-glutamic acid (PGA) both served as reservoirs for the loading and sustained release of the above β^3^-peptide [248]. Moreover, CH/HA films exhibited inherent antifungal and antibiofilm activity in vitro and in vivo, which may be attributed to CH being a weak polycation. Nevertheless, the antifungal properties of both types of coatings were improved substantially when the β^3^-peptide was incorporated. Catheter segments coated with β^3^-peptide-loaded CH/HA or PLL/PGA films displayed efficient antifungal effects on planktonic *C. albicans* and substantially reduced formation of *C. albicans* biofilms both in vitro and in vivo using a rat model of central venous catheter infection [248].

### 2.6. α-Peptide/β^3^-Peptide Hybrids

Inspired by previous work concerning design of antibacterial helical β^3^-peptides [238], the group of Gellman explored α-peptide/β^3^-peptide hybrids, consisting of L-Lys and L-Leu as well as the constrained (via five-membered rings) β^3^-amino acids ACPC and APC in a 1:1 ratio, and with an alternating design (also referred to as α/β-peptides). Such peptidomimetics typically adopt either a so-called 11-helix (i.e., 11 atoms in the hydrogen-bonded ring; ca. 3 residues per turn) or a 14/15-helix (ca. 4.5 residues per turn) [249]. Two 15-residue sequence isomers were designed to display an amphipathic 11-helix (i.e., H-APC-Leu-ACPC-Lys-ACPC-Leu-APC-Leu-ACPC-Lys-ACPC-Leu-APC-Leu-ACPC-NH_2_) or an amphipathic 14/15-helix (i.e., H-APC-Leu-ACPC-Leu-APC-Leu-ACPC-Lys-ACPC-Leu-ACPC-Lys-ACPC-Leu-APC-NH_2_), respectively. For comparison a scrambled sequence (i.e., H-ACPC-Leu-APC-Lys-APC-Leu-ACPC-Lys-APC-Leu-ACPC-Leu-ACPC-Leu-ACPC-NH_2_), devoid of amphipathicity in either of these conformations, was included. Intriguingly, the isomer designed to adopt an amphipathic 14/15-helical conformation proved to be least active among the three α/β-peptide isomers. Noticeably, the scrambled analog, designed not to possess amphipathicity in either helical conformation, was found to be the most potent isomer against *E. coli*, while the putative 11-helical isomer exhibited superior activity against Gram-positive *E. faecium* and *S. aureus* strains. This is contradictory to the trend seen for β^3^-peptides, for which an amphipathic helical structure was essential for high antibacterial activity [238]. However, the most striking discovery was that the scrambled isomer was non-hemolytic at 15- to 30-fold higher concentrations than the two isomers designed to adopt helical conformations [249]. In a subsequent study, an alternative scrambled sequence (H-APC-Leu-APC-Leu-ACPC-Lys-ACPC-Leu-ACPC-Leu-APC-Leu-ACPC-Lys-ACPC-NH_2_) with a more even distribution of cationic residues along the sequence was investigated [241], while the original scrambled sequence displayed “longitudinal” amphipathicity with cationic residues clustered towards the N-terminus [249]. However, similar activity profiles were seen for these two hybrids (MICs typically in the range 6.3–12.5 µg/mL). Another α/β-peptide (i.e., H-APC-Leu-ACPC-Leu-APC-Leu-ACPC-Leu-APC-Leu-ACPC-Lys-ACPC-Leu-APC-NH_2_), designed to achieve more pronounced amphipathicity in a 14/15-helical conformation, displayed more potent activity against pathogenic bacteria (MICs of 12.5–25 µg/mL), but remained hemolytic at the MIC [250]. Examination of a more polar set of isomeric α/β-peptides displaying triple Leu → Ala substitutions (as compared to the original sequences) revealed that such changes gave rise to different effects on the relative antibacterial potency. For the pair designed to be amphipathic in the 11-helical conformation the Leu → Ala substitutions led to slightly lower activity against Gram-positive strains, while the effect for the putative amphipathic 14/15-helical pair was opposite, with the polar analog having generally improved activity (MICs of 3.1–12.5 µg/mL). Noticeably, the polar analog with an 11-helical amphipathic design was devoid of hemolytic activity even at 400 µg/mL, while the polar 14/15-helical compound had an HC_10_ of 100 µg/mL. By contrast, the Leu → Ala mutations in the scrambled isomer conferred a dramatic loss of activity [250]. Hence, design strategies toward antimicrobial oligomers with favorable cell selectivity should be extended to include peptidomimetics not displaying amphipathicity, albeit the outcome, in terms of whether a desirable overall activity profile may be achieved, appears to depend on the hydrophobicity of the compounds. Table 7 shows the activity profiles for some of the most active α-peptide/β^3^-peptide hybrids.

### 2.7. AApeptides

AApeptides were first described by Cai et al. in 2011 [251]. They are oligomers of *N*-acetylated *N*-aminoethylamino acid units, which are derived from the chiral peptide nucleic acid (PNA) backbone. AApeptides are divided into two main subclasses, namely α-AApeptides [252] and γ-AApeptides [253], depending on the position of the side chain (Figure 1). Other subclasses include lipidated linear α-AApeptides [254], lipidated cyclic α-AApeptides [255], lipidated linear γ-AApeptides [256], cyclic γ-AApeptides [257], lipidated cyclic γ-AApeptides [258], sulfono-γ-AApeptides [259,260], and combinations of these [260]. AA-peptides are enzymatically stable, display excellent antimicrobial activity and show promise in many other biological applications such as cancer, Alzheimer’s disease and biomaterial science [261]. AApeptides have recently been reviewed [261,262,263].

#### 2.7.1. Synthesis

A general strategy for the synthesis of AApeptides is as follows: (i) Synthesis of AApeptide building blocks; (ii) Assembly on solid-phase; (iii) Optional lipidation and/or cyclisation on solid phase; (iv) Cleavage from resin, HPLC purification and lyophilisation [264].

#### 2.7.2. Synthesis of α-AApeptides

**Scheme 14:**
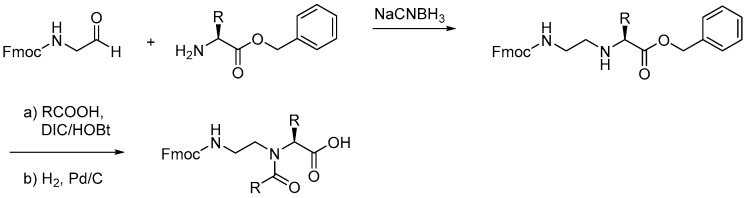
General synthesis of α-AApeptide building blocks [264].

α-AApeptides are synthesized by SPS using Fmoc-protected α-AApeptide building blocks (Scheme 14). These building blocks are prepared by reductive amination involving the benzyl ester of the amino acid, Fmoc-glycine aldehyde and sodium cyanoborohydride. Following acylation of the intermediate with a carboxylic acid (e.g., 3-phenylpropionic acid), the benzyl protecting group is removed by hydrogenation to give the desired building block [264]. The α-AApeptide sequence is then assembled by using standard Fmoc-based SPS with DIC/HOBt as coupling reagents.

#### 2.7.3. Synthesis of γ-AApeptides

γ-AApeptides are synthesized by SPS using *N*-Alloc γ-AApeptide preformed building blocks [265], which may be prepared by two different routes; route A is used when the side chain (R) is unprotected, while route B is employed when the side chain requires protection by an acid-labile group.

**Scheme 15:**
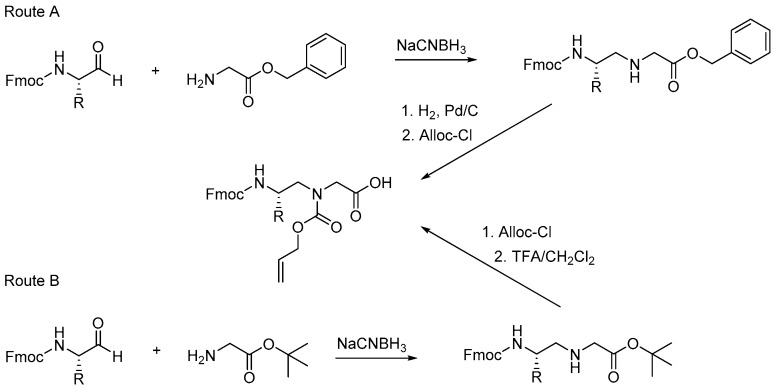
Synthesis of γ-AApeptide building blocks by two different routes.

**Scheme 16:**
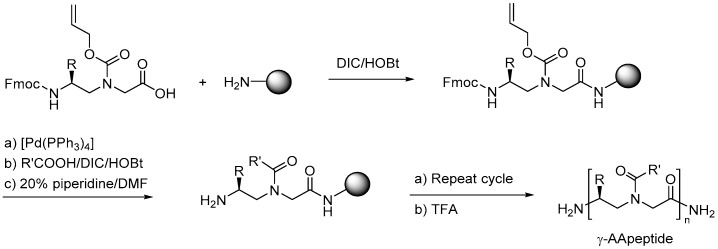
γ-AApeptide oligomer assembly via Fmoc-based SPS; adapted from Teng et al. [261].

In route A (Scheme 15), the benzyl ester of glycine, an Fmoc-protected amino aldehyde and sodium cyanoborohydride are condensed, followed by hydrogenolysis and treatment with Alloc-Cl to give the protected γ-AApeptide building block. The first step of route B involves a reductive amination of glycine *t*-butyl ester with an Fmoc-protected amino aldehyde and sodium cyanoborohydride. Following reaction with Alloc-Cl, the *t*-butyl group is cleaved by 50% TFA in DCM. Alternatively, the Alloc group may be cleaved and acylated to generate a number of different backbone scaffolds, which can be employed in SPS [265]. The γ-AApeptide sequence is then assembled by using Fmoc-based SPS as shown in Scheme 16.

It is worth noting that both α-AApeptides and γ-AApeptides may also be synthesized by submonomer approaches that circumvent the need for preparation of building blocks [266]. However, these approaches require many additional steps and results in significantly lower yields when synthesizing longer sequences [263].

#### 2.7.4. Antimicrobial Activity

##### α-AApeptides and γ-AApeptides

Padhee and co-workers first reported on the antimicrobial activity of α-AApeptides [252]. The authors studied α-AApeptides consisting of one to six cationic/hydrophobic building blocks (i.e., *N*^α^-(2-aminoethyl)-*N*^α^-(3-phenylpropanoyl)lysine), against different Gram-positive and Gram-negative bacteria. They found that the compound containing six of these α-AApeptide residues exhibited the best activity against *S. epidermidis* (MIC of 4.6 µg/mL) and *B. subtilis* (MIC of 1 µg/mL). Furthermore, HC_50_ was found to be larger than 250 µg/mL.

**Figure 13:**
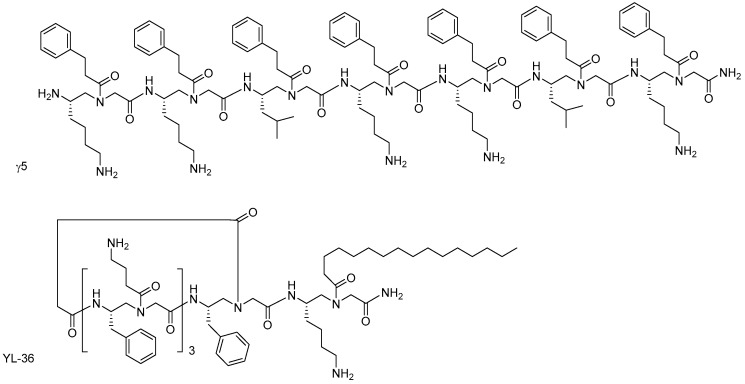
Most active compounds found in the studies by Niu [253] and Li [258].

The antimicrobial activity of γ-AApeptides was first reported by Niu et al. [253]. They studied γ-AApeptides of different lengths (n = 3, 5, 7), and found that seven γ-AA residues are necessary for potent activity. The most potent compound proved to be γ5 (Figure 13), which displayed MICs of 2–6.3 µg/mL against different MDR Gram-positive and Gram-negative bacteria, while HC_50_ was 300 µg/mL.

Besides antibacterial activity, a number of other studies have revealed a potential for mimicking other biologically active peptides. Thus, a ten-residue γ-AApeptide mimic of Tat peptide (GRKKRRQRRRPQ) was able to bind to HIV TAR RNA and BIV TAR RNA with comparable selectivity and specificity to that of the Tat peptide [267]. Furthermore, γ-AApeptide mimics of RGD peptides (including ^64^Cu-labelled compounds) [268], and of fMLF [269] (a selective formyl peptide receptor agonist) have been reported. In addition, γ-AApeptides have been shown to disrupt Aβ aggregation in Alzheimer’s disease [270], and to have potential applications in biomaterial science [271].

##### Lipidated Linear AApeptides and Lipocyclic AApeptides

Lipidation is a well-known approach for increasing the interaction between an antimicrobial peptide and the bacterial envelope. A series of lipidated α-AApeptides, carrying two-six residues and a fatty acid attached to the *C*-terminus were reported by Hu et al. [254]. The most active compound, consisting of five *N*-(4-aminobutanoyl)-*N*-(2-aminoethyl)-l-phenylalanyl trimeric units and carrying a C_16_ lipid tail, showed MICs of 4–12 µg/mL against different Gram-positive and Gram-negative bacteria, while HC_50_ was 250 µg/mL.

Daptomycin and colistin are two naturally occurring cyclic lipopeptide antibiotics, used for treatment of Gram-positive and Gram-negative infections, respectively. In recent years, considerable efforts have aimed at designing less toxic antimicrobial mimics of these compounds. The antimicrobial and hemolytic activity of six cyclic lipo-α-AApeptides, containing 3–5 residues and a C_6_, C_12_ or C_16_ lipid tail, were investigated by Padhee et al. [255]. The most potent compound showed MICs of 1–10 µg/mL against Gram-positive and Gram-negative bacteria and an HC_50_ of 150 µg/mL. The authors also conducted mechanistic studies, and this compound was able to permeate MRSA membranes (as indicated by staining with 4′,6-diamidino-2-phenylindole dihydrochloride and propidium iodide), indicating loss of membrane potential or lysis. Furthermore, the authors showed that the lead compound can mimic host-defense peptides by antagonizing Toll-like receptor 4 (TLR4)-induced responses via suppressing secretion of proinflammatory cytokines.

In a follow-up study [258], the authors showed that lipidated cyclic γ-AApeptides display potent antibacterial activity against clinically relevant bacteria. The best compound, YL-36 (Figure 13), showed MICs of 1–3 µg/mL against different Gram-positive and Gram-negative bacteria, while HC_50_ was 100 µg/mL. Increasing the ring size did not improve the antimicrobial activity. The above lipidated cyclic γ-AApeptides have also been tested against planktonic cells and biofilms of *S. epidermidis* and *P. aeruginosa*. In comparison with ciprofloxacin they performed equally well in preventing formation of biofilm of Gram-positive and Gram-negative bacteria [272]. The authors also studied how lipidated cyclic γ-AApeptides disrupt bacterial membranes by electron paramagnetic resonance, which suggested that they act through a carpet mechanism [273].

Sulfono-γ-AApeptides are peptidomimetics that are oligomers of *N*-sulfono-acylated *N*-aminoethyl amino acids [259]. Li et al. synthesized 15 analogs displaying different length and distribution of hydrophobic/cationic residues. Longer oligomers, containing additional cationic and hydrophobic groups, were more potent than shorter ones. The most active compound was an eight-mer, which showed MIC values of 2–4 µg/mL against Gram-positive and Gram-negative bacteria as well as an HC_50_ of 100 µg/mL. This compound killed MRSA and *E. coli* within 30 and 60 min, respectively, when applied at 12 × MIC. X-ray crystallography and 2D NMR analysis suggested that eight-mers of sulfono-γ-AApeptides adopt a well-defined helical structure in solution.

Recently, She et al. reported a related subclass of peptidomimetics (1:1 sulfono-γ-AA heterogeneous peptides) with antibacterial activity [260]. The best compound showed an MIC of 3 µg/mL against MRSA and 4 µg/mL against *E. coli* and *K. pneumonia*. Furthermore, the authors found a HC_50_ above 250 µg/mL. Small-angle scattering experiments suggested that this compound adopts a helical structure. Time-kill experiments showed that the lead compound kills MRSA in 2 h at 16 × MIC. Table 8 shows the activity profiles for selected AApeptides.

### 2.8. Oligo-Acyl-Lysyl Peptidomimetics (OAKs)

Mor et al. introduced peptidomimetic sequences consisting of alternating acyl chains and cationic amino acids, termed oligo AQs (OAQs), where A represents a fatty acid of variable length while Q is a cationic amino acid. However, since the cationic amino acid often is lysine, they are now known as OAKs [275]. For reviews see Rotem and Mor [276] and Mor [277]. This class of peptidomimetics is composed of a small number of building blocks referred to as α_i_ (acyl-lysyl) or β_i_ (lysyl-acyl-lysyl) subunits where i specifies the number of carbons in the acyl moiety. OAKs may display broad-spectrum antimicrobial activity against MDR bacteria [278,279], selectivity against Gram-negative bacteria [280] as well as *S. aureus* [281]. Furthermore, OAKs displaying potent activity against *Helicobacter pylori* [282,283] and malaria parasites [284,285] have been reported.

#### 2.8.1. Synthesis

**Figure 14:**
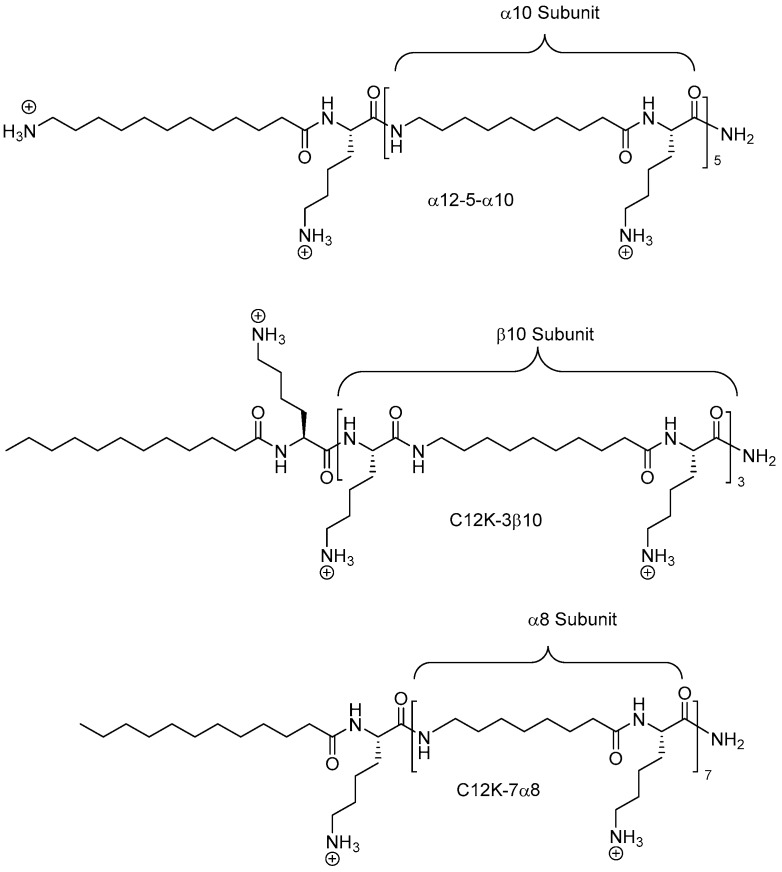
Representative OAK structures.

OAKs are synthesized by standard Fmoc-based SPPS [286], e.g., by using DIC/HOBt or HBTU/HOBt as coupling reagents. Starting from an H-Lys(Boc)-4-methylbenzhydrylamine resin, the appropriate Fmoc-protected ω-amino fatty acid, e.g., *N*-(9-fluorenylmethyloxycarbonyl)-10-amino-decanoic acid, is coupled to the *N*^α^-group of the resin-bound C-terminal Lys residue. The Fmoc group is then removed with 20% piperidine in DMF, yielding a basic α_10_ subunit. If a basic β_10_ subunit is required another Fmoc-Lys(Boc)-OH is coupled and deprotected. The described process is repeated until the desired sequence is obtained. The product is then cleaved with 95% aqueous TFA using standard SPPS procedures, purified by preparative HPLC and lyophilized. In Figure 14 representatives of different subtypes of OAKs are depicted.

#### 2.8.2. Antimicrobial Activity

Radzishevsky et al. described 16 OAKs [275] comprising the seven butyroyl-based C_4_K(1-7)α_4_, the seven octanoyl-based C_8_K(1-7)α_8_ OAKs as wells as two substituted OAKs (C_12_K-7α_4_ and C_12_K-7α_8_). The best compound was C_12_K-7α_8_, which showed potent activity against most Gram-negative bacteria tested (MICs of 1.6–12.5 µM), including *Pseudomonas*, *Acinetobacter* and *Klebsiella* spp. At 6 × MIC, this compound was able to reduce *E. coli* bacterial populations from 10^6^ to <50 CFU/mL within 1 h, which is faster than both imipenem and ciprofloxacin. Furthermore, C_12_K-7α_8_ displayed virtually no hemolytic activity.

The experimental conditions that enhance the potency of C_12_K-7α_8_ against *E. coli*, *Listeria monocytogenes* and *S. aureus* were reported by Goldfeder et al. [287]. They found that the potency was proportionally affected by pH changes and subsided essentially throughout a wide range of salt concentrations and temperature values, whereas anti-staphyloccocal activity was relatively more vulnerable. The optimal incubation conditions resulted in a 16- to 32-fold reduction of the MIC value.

Livne et al. [279] determined the MIC of C_12_K-7α_8_ in combination with different antibiotics against MDR strains of *E. coli*, and found that subMIC levels of C_12_K-7α_8_ acted synergistically with several antibiotics. Single-dose administration of erythromycin co-encapsulated in C_12_K-7α_8_-based cochleates was effective in systemic treatment of neutropenic mice infected with lethal inoculums of MDR *E. coli*. In a follow-up study, Sarig et al. [288] compared the encapsulation efficiency in different lipid mixtures with C_12_K-7α_8_ in the presence and absence of erythromycin. They showed that the in vivo activity of C_12_K-7α_8_ and erythromycin against MDR bacteria can be enhanced significantly by incorporating the drugs into cochleates as compared to their activity in the absence of lipid.

Epand et al. [289] used light microscopy to screen for cochleate formation in several OAK-lipid systems including C_12_K-7α_8_, while freeze-fracture electron microscopy was employed to assess their morphological features and size. They found that in OAK-based lipid systems, cochleate formation is enhanced by dehydration of the membrane interface and a high melting temperature.

A structure-activity study of 103 OAKs, designed to assess the importance of charge and hydrophobicity was reported by Radzishevsky [290]. They systematically modified both the acyl length (butanoyl, octanoyl, or dodecanoyl) and the number of α-subunits (1–9). The most active compound, C_12_-2β_12_, displayed an MIC of 3.1 µM and 1.6 µM against *E. coli* and *S. aureus*, respectively, while HC_50_ was 17 µM. The authors found that the butyryl- and octanoyl-based series were non-hemolytic and displayed activity when the charge was equal to or above +5 and the hydrophobicity corresponded to at least 45% acetonitrile in the eluent at peak elution on a C_18_ HPLC column. For the dodecanoyl-based OAKs, antibacterial activity was achieved already with the small compound C_12_K-α_12_ (charge +2 and hydrophobicity ~56% MeCN) that had modest hemolytic activity. All longer derivatives showed a strong hemolytic activity and were inactive (MIC above 50 µM). The authors also noted that for a potent antibacterial activity (<3.1 µM) against *E. coli* a sufficient hydrophobicity (~50% MeCN) and charge of 6.5 ± 2 are required. For *S. aureus* these numbers are the 48% and 4 ± 1, respectively.

Livne et al. [278] reported a structure-activity study of three series of OAKs, all having a lauroyl-lysyl at the N-terminus and 1–5 α- or β-subunits. Generally, the more hydrophobic α_10_-OAKs showed increased hemolytic properties and a tendency to aggregate with increasing length. The less hydrophobic β_10_ series showed significantly less hemolytic activity and aggregation. The most active compound, C_12_K-3β_10_, displayed MICs of 3.1–6.2 µM against *E. coli* and *P. aeruginosa* and 1.6–12.5 µM against *A. baumannii* and *Staphylococcus* spp. Furthermore, HC_50_ was >100 µM. The mode of action of C_12_K-3β_10_ was investigated, and this showed that its effects on *E. coli* and *S. aureus* are bactericidal and bacteriostatic, respectively.

The cell-wall interactions and the selective bacteriostatic activity of C_12(ω-7)_K-β_12_, which is 10-fold more potent against Gram-positive bacteria, have been studied by isothermal titration calorimetry and permeabilization assays [291]. The authors found that in Gram-positive bacteria, C_12(ω-7)_K-β_12_ accumulates in the small periplasm followed by electrostatic interactions. However, C_12(ω-7)_K-β_12_ does not cross the outer membrane of Gram-negative bacteria, but acts by depositing on the LPS layer. A study by Kaneti et al. [281] describes the anti-staphylococcal synergistic activity observed between subinhibitory concentrations of C_12(ω-7)_K-β_12_ and several antibiotics (e.g., ciprofloxacin, gentamicin and β-lactams) targeting distinct cytoplasmic and extracellular molecular entities. These OAKs were able to improve the potency of oxacillin and piperacillin from MICs of 256 µg/mL to 0.256 µg/mL, and it was suggested that transient membrane depolarization was the reason. In another study, similar results were obtained with *E. coli*, where C_12(ω-7)_K-β_12_ at subinhibitory concentrations enhanced the activity of erythromycin three orders of magnitude [292].

The antibacterial mechanism of action of a single OAK may vary among intra- and interspecies strains. Sarig et al. [293] investigated the mechanism of action of C_16(ω-7)_-KKC_12_-K_amide_, against a multispecies and multi-strain panel of bacteria. Mechanistic comparison between equally sensitive and related strains showed that death of one strain resulted from C_16(ω-7)_-KKC_12_-K_amide_ being able to disrupt the cell membrane permeability barrier, whereas the death of a related strain resulted from the direct interference of C_16(ω-7)_-KKC_12_-K_amide_ with DNA functions, without detectable membrane damage.

Acyl-lysyl oligomers have shown activity against the gastric pathogen *Helicobacter pylori*. Makobongo et al. [282] screened fived OAKs for activity against *H. pylori* strain G27. The most active compound was C_12_K-2β_12_, which displayed MIC and MBC (minimum bactericidal concentration) in the ranges of 6.5–26 µM and 14.5–90 µM, respectively, across the six strains tested. At 2 × MBC, C_12_K-2β_12_ completely killed *H. pylori* after 6–8 h of incubation in liquid cultures. Furthermore, the compound displayed only modest hemolytic activity (4–8%) at 80 µM. In a follow-up study [283], the authors showed that C_12_K-2β_12_ rapidly permeabilizes the bacterial membrane and crosses the bacterial membranes into the cytoplasm and binds tightly to bacterial DNA, RNA, and proteins.

Zaknoon et al. [284] reported the screening of more than 50 OAKs against the virulent malaria parasite *Plasmodium falciparum*. The most active compound was C_12_K-2α_8_, which showed an IC_50_, of 0.3 µM and no hemolytic activity at 150 µM. Furthermore, C_12_K-2α_8_ significantly affected parasite growth in *Plasmodium vinckei*-infected mice. Table 9 shows the activity profiles for selected OAKs.

## 3. Conclusions and Outlook

In the present review, recent advances in development of antimicrobial peptidomimetics as potential drugs have been discussed with respect to accessibility via chemical synthesis and optimization strategies for obtaining compounds with an appropriate pharmacological profile. The key hurdles with peptidomimetics as therapeutics are: (i) availability; (ii) toxicity; (iii) bioavailability; and (iv) drug approval regulations (which applies to all antibiotics).

In order for synthetic peptidomimetics to be considered as actual drug entities they should be readily prepared in large scale, e.g., via assembly on solid phase by simple methods involving as few steps as possible. Hence, commercial availability and/or easy preparation of the starting materials constitute important factors when considering the practical utility of a class of peptidomimetics as a source of actual drugs; e.g., OAKs, peptoids and their peptide hybrids may be prepared efficiently from cheap starting materials, while assembly of β^3^-peptides and AApeptides require more elaborate syntheses of building blocks. Also, simple designs consisting of a very few different residues may facilitate up-scaling by allowing for combination of solution-phase and solid-phase methods, e.g., via block syntheses.

Although many peptidomimetics exhibit excellent antimicrobial potency in vitro (i.e., MICs below 2 µM) with rapid killing of bacteria they may exert unexpected side effects when tested via in vivo animal models. Thus, peptides and peptidomimetics designed for optimal antimicrobial activity often possess undesired safety profiles since the properties promoting efficient bactericidal effect also to some extent confer increased toxicity toward mammalian cells including immediate immunogenic responses. These characteristics may explain why no peptidomimetics have reached the market yet.

Typically, chemical modification toward lower toxicity involves adjustment of hydrophobicity to below the critical threshold, and the conformation toward the most favorable secondary structure (appears to depend on the subclass). However, another key challenge is to deliver the antimicrobial drug to its target with high efficacy and few side effects, which may be addressed by development of appropriate formulations. This may involve e.g., conjugation to charge-reducing prodrug moieties [294] and/or shielding of the optimized molecule by PEGylation [295] or encapsulation in biocompatible polymer-based nanoparticles [296,297,298,299], which all may contribute to improvement of the efficacy-safety profile due to a lower required dose because of improved distribution into infected tissue.

Another promising prospective application for peptidomimetics is as potentiators of other antibiotics e.g., via membrane permeabilization conferring enhanced uptake of antibiotics thereby circumventing antimicrobial resistance arising from upregulated bacterial efflux or membrane modifications. Also, they may be employed for bacterial delivery of other antibiotics, e.g., antisense PNA oligomers. Peptidomimetics of AMPs display a number of other interesting activities such as anticancer activity [300], antibiofilm activity [184] and anti-inflammatory effects [223].

In order for antibiotic development to be attractive for the pharmaceutical industry the following are needed: (i) shorter time to market, and (ii) prolonged market exclusivity [20]. To this end, new regulatory incentives may stimulate the development of novel antibiotics. For example, the GAIN ACT (Generating Antibiotic Incentives Now), by which new antibiotics may be designated as QIDPs (Qualified Infectious Disease Products) if they target one or more of 20 critical pathogens. When these criteria are met such antibiotics may receive prolonged market exclusivity for up to 12 years. Another incentive is the Century Cures Act of 2015, allowing approval of new antibiotics based on studies in limited patient populations rather than full clinical trials [20].

In conclusion, recent progress in optimization of antimicrobial peptidomimetics combined with an increased focus on appropriate formulation for improved delivery may unleash the potential of antimicrobial peptidomimetics as future antibiotics.

## Figures and Tables

**Table 1:** AMPs in clinical trials (Phase I–III).

AMP	AMP source	Target	Phase	Company	Admin.	Refs.
NVB-302	Lantibiotic	*C. difficile*	I *	Novacta	Oral	[62]
hlf1-11	Lactoferricin analog	Infection following transplantation	I/II	AM-Pharma	I.v.	[63]
Wap-8294A2	Lysobactor spp.	G^+^ bacteria (VRE and MRSA)	I/II	aRigen	Top.	[64]
C16G2	Synthetic peptide	Prevention of tooth decay caused by *Streptococcus mutans*	II	C3 Jian Inc.	Mouth wash	[65,66]
IMX942 (SGX942)	Indolicidin analog	Immunomodulation during oral mucositis	II	Soligenix	Oral rinse	[67]
DPK-060	Human protein kininogen	Acute external otitis	II	Pergamum AB	Ear drops	[68]
PXL01	Human lactoferrin analog	Surgical adhesion prevention	II	Pergamum AB	Top.	[69]
Brilacidin	Defensin analog	Radiation-induced oral mucositis in patients with head and neck cancer	II	Cellceutix Corp	Oral rinse	[70]
PAC113	Histatin analog	Oral candidiasis in HIV seropositive patients	II	Pacgen Biopharmaceuticals Corp.	Mouth rinse	[71]
POL7080	Protegrin analog	Ventilator-associated *P. aeruginosa* pneumonia	II ***	Polyphor Ltd.	I.v.	[72,73]
LTX-109 (Lytixar)	Synthetic peptidomimetic	Nasal decolonisation of MRSA G^+^ skin infections	IIa *	Lytix Biopharma	Nasal Top.	[74,75,76]
		Diabetic foot ulcer	PC			
OP-145	LL-37	Chronic middle ear infection	II *	Dr. Reddy’s Research	Ear drops	[77,78]
LL-37	Human cathelicidin	Leg ulcer	IIb	ProMore Pharma	Top.	[79,80]
Novexatin^®^ (NP213)	Cyclic cationic peptide	Fungal nail infection	IIb	Novabiotics	Top.	[81]
p2TA (AB 103)	Synthetic peptide	Necrotizing soft tissue infections	III	Atox Bio Ltd	I.v.	[82]
Iseganan	Protegrin analog	Ventilator-associated pneumonia	III **	IntraBiotics Pharmaceuticals	Top.	[83,84]
Locilex (MSI-78)	Magainin analog (Pexiganan)	Infections of diabetic foot ulcers	III **	Dipexium Pharmaceuticals	Top.	[85]
Omiganan (CLS001)	Indolicidin derivative	CAUTI caused by *S. aureus*;	IIIb **	Mallinckrodt	Top.	[86]
		Topical skin antisepsis, rosacea;	III	Mallinckrodt		
		Vulvaryl intraepithelial neoplasia, acne, atopic dermatitis	II	Cutanea Life Sciences		
Suroto- mycin	Cyclic lipopeptide	*C. difficile* (diarrhea)	III *	Cubist Pharmaceuticals/Merck	Oral	[87]
Ramoplanin (NTI-851)	Actinoplanes spp	VRE (G^+^)	III	Nano-therapeutics	Oral	[88,89]
		*C. difficile* (G^+^)	II			

* Completed; ** Failed; *** Terminated; Top.—topical; I.v.—intravenous.

**Table 2:** AMPs in preclinical trials.

AMP	AMP Source	Target	Phase	Company	Admin.	Refs.
MU1140	*Streptococcus mutans* (lantibiotic)	G^+^ bacteria (MRSA, *C. difficile*)	PC	Oragenics	Not specified	[90,91]
HB1345	Lipopeptide	Broad-spectrum antibiotic, acne	PC	Helix Biomedix	Top.	[92]
Novarifyn (NP432)	Synthetic antimicrobial	MRSA, *P. aeruginosa*, *C. difficile*, *A. baumannii*, *E. coli*	PC	Novabiotics	Top.	[93]
Arenicin (AP139)	Lugworm Arenicol marina	G^−^ bacteria, UTI	PC	Adenium Biotech	Not specified	[94,95]
AP138	Arenicin analog	MRSA implant infections	PC	Adenium	Not specified	[96]
AP114	Arenicin analog	*C. difficile*	PC	Adenium	Not specified	[96]
Avidocin and purocin	Modified R-type bacteriocin	G^+^ and G^−^ bacteria	PC	AvidBiotics	Oral	[97,98]

Top.—topical.

**Table 3:** Antimicrobial activity and cytotoxicity of representative peptoids.

Compound	MIC Values Cytotoxicity				
	*S. aureus*	*E. coli*	*P. aeruginosa*	EC_50_^a^/HC_50_^b^	Refs.
H-(*N*hArg-*N*spe-*N*spe)_4_-NH_2_	1 µM	6 µM	13 µM	12 µM ^a^	[179]
H-(*N*Lys)_4_(*N*hTrp)_4_-*N*Ile-NH_2_	8 µg/mL	8 µg/mL	4 µg/mL	128 µg/mL ^c^	[162,177]
c(*N*ap-*N*dpe)_3_	3.9 µg/mL	ND	7.8 µg/mL	>250 µg/mL ^b^	[190]

^a^ concentration which is toxic to 50% of HEPG2 cells; ^b^ concentration giving rise to 50% hemolysis; ^c^ concentration which generates 10% hemolysis of red blood cells.

**Table 4:** Antimicrobial activity of representative AMPs containing peptoid residues.

Compound	MIC (in µM) Hemolysis				
	*S. aureus*	*E. coli*	*P. aeruginosa*	HC_50_	Ref.
Pis-1[NkG] ^a^	4	2	4	>200	[197]
KLW-f ^b^	1	2	1	>100	[192]
L_8_K_9_W_1_-*N*Leu ^c^	2	2	1	>100	[194]
STPk ^d^	1	1	2	>200	[196]

^a^ Pis-1[NkG] = FFHHIFR-*N*Lys-IVHVGKTIHRLVTG; ^b^ KLW-f = KWKKLLKK-*N*Phe-LKL-*N*Phe-KKLLK-NH_2_; ^c^ L_8_K_9_W_1_-*N*Leu = KWKKLLKK-*N*Leu-LKL-*N*Leu-KKLLK-NH_2_; ^d^ STPk = KKF-*N*Lys-WWW-*N*Lys-FKK-NH_2_

**Table 5:** Antimicrobial and hemolytic activity of representative peptide-peptoid hybrids.

Compound	MIC (in µM or µg/mL) Hemolysis			
	*S. aureus*	*P. aeruginosa*	HA ^a^	Ref.
IK-*Ns*sb-*N*Lys-VRK-*N*ssb-NH_2_	3.1 µg/mL	11.8 µg/mL	-	[202]
*N*Lys-*N*Lys-W-*N*smb-IKRW-NH_2_	9.2 µg/mL	5.7 µg/mL	-	[202]
*N*nal-*N*mphe-*N*nal-*N*nle-KKKKK-NH_2_	1.6 µM	12.5 µM	3%	[204]
KKKK-*N*nal-*N*mphe-*N*nal-*N*nle-NH_2_	1.6 µM	6.25 µM	6%	[204]

^a^ % hemolysis at 50 µM.

**Table 6:** Antimicrobial and hemolytic activity of representative α-peptide/β-peptoid hybrids.

Compound	MIC (in µM) Hemolysis (in µM)				
	*S. aureus*	*E. coli*	*P. aeruginosa*	HC_10_	Refs.
Ac-[Lys-β*N*spe]_8_-NH_2_	37	9.1	4.7	>500	[216]
Ac-[hArg-β*N*Phe]_6_-NH_2_	5.9	1.5	-	>500	[216,217]
Ac-[hArg-β*N*spe]_6_-NH_2_	2.8	2.8	-	>500	[216,217]
Ac-[Lys-β*N*Phe-hArg-β*N*Phe]_4_-NH_2_	32	1	-	-	[120]
cHex-[hArg-β*N*spe-Lys-β*N*spe]_4_-NH_2_	1	2	-	600 ^a^	[218]
Ac-[Lys-β*N*(4-F-Phe)]_8_-NH_2_	0.8	3.2	1.6	>150	[219]

^a^ HC_50_.

**Table 7:** Antimicrobial and hemolytic activity of representative α-peptide/β^3^-peptide hybrids.

Compound	MIC (in µg/mL) Hemolysis (in µg/mL)			
	*S. aureus*	*E. coli*	HC_10_	Ref.
H-ACPC-Leu-APC-Lys-APC-Leu-ACPC-Lys-APC-Leu-ACPC-Leu-ACPC-Leu-ACPC-NH_2_	12.5	6.3	100	[250]
H-APC-Leu-ACPC-Lys-ACPC-Ala-APC-Ala-ACPC-Lys-ACPC-Ala-APC-Leu-ACPC-NH_2_	12.5	6.3	>400	[250]

**Table 8:** Antimicrobial and hemolytic activity of selected AApeptides (in µg/mL).

	MIC Hemolysis					
Compound type	ID	MRSA	*P. aeruginosa*	*K. pneumonia*	HC_50_	Ref.
α-AApeptide	α4	4	8	8	>250	[274]
γ-AApeptide	γ-5	5	ND	5	300	[253]
Cyclic γ-AApeptide	HW-B-13	1	8	8	100	[257]
Lipo-γ-AA	13	3	3	3	>500	[256]
Cyclic lipo-γ-AA	YL-36	2	5	3	100	[258]
Sulfono-γ-AApeptide	7	2	4	ND	100	[259]
1:1 α/sulfono-γ-AApeptide	6	3	4	4	>250	[260]

**Table 9:** Antimicrobial and hemolytic activity of selected OAKs (in µM).

	MIC Hemolysis				
Compound	*S. aureus*	*E. coli*	*P. aeruginosa*	HC_50_	Ref.
C_12_K-7α_8_	50	1.6	6.2	>100	[275]
C_12_-2β_12_	1.6	3.1	ND	17	[290]
C_12_K-3β_10_	1.6–12.5 ^a^	3.1–6.2 ^b^	3.1–6.2 ^c^	>100	[278]

^a^ *Staphylococcus* spp. (n = 9), ^b^ (n = 13), ^c^ (n = 11).
